# Mitigating nitrogen losses with almost no crop yield penalty during extremely wet years

**DOI:** 10.1126/sciadv.adi9325

**Published:** 2024-02-28

**Authors:** Wenfeng Liu, Mengxue Li, Yuanyuan Huang, David Makowski, Yang Su, Yawei Bai, Bernhard Schauberger, Taisheng Du, Karim C. Abbaspour, Kun Yang, Hong Yang, Philippe Ciais

**Affiliations:** ^1^ State Key Laboratory of Efficient Utilization of Agricultural Water Resources, Beijing 100083, China.; ^2^ National Field Scientific Observation and Research Station on Efficient Water Use of Oasis Agriculture in Wuwei of Gansu Province, Wuwei 733000, China.; ^3^ Center for Agricultural Water Research in China, College of Water Resources and Civil Engineering, China Agricultural University, Beijing 100083, China.; ^4^ Key Laboratory of Ecosystem Network Observation and Modeling, Institute of Geographic Sciences and Natural Resources Research, Chinese Academy of Sciences, Beijing 100101, China.; ^5^ UMR Applied Mathematics and Computer Science (MIA518), INRAE AgroParisTech, Université Paris-Saclay, Palaiseau, France.; ^6^ UMR ECOSYS, INRAE UVSQ, Université Paris-Saclay, 91190 Gif-sur-Yvette, France.; ^7^ Département d'Informatique, École Normale Supérieure - PSL, 75005 Paris, France.; ^8^ Laboratoire des Sciences du Climat et de l’Environnement, LSCE/IPSL, CEA-CNRS-UVSQ, Université Paris-Saclay, Gif-sur-Yvette, France.; ^9^ University of Applied Sciences Weihenstephan-Triesdorf, Department of Sustainable Agriculture and Energy Systems, Am Staudengarten 1, 85354 Freising, Germany.; ^10^ Potsdam Institute for Climate Impact Research (PIK), Member of the Leibniz Association, 14473 Potsdam, Germany.; ^11^ 2w2e Environmental Consulting GmbH, Mettlenweg 3, Dübendorf, 8600 Zürich, Switzerland.; ^12^ Department of Earth System Science, Ministry of Education Key Laboratory for Earth System Modeling, Institute for Global Change Studies, Tsinghua University, Beijing100084, China.; ^13^ National Tibetan Plateau Data Center, State Key Laboratory of Tibetan Plateau Earth System and Resource Environment, Institute of Tibetan Plateau Research, Chinese Academy of Sciences, Beijing 100101, China.

## Abstract

Climate change–induced precipitation anomalies during extremely wet years (EWYs) result in substantial nitrogen losses to aquatic ecosystems (N_w_). Still, the extent and drivers of these losses, and effective mitigation strategies have remained unclear. By integrating global datasets with well-established crop modeling and machine learning techniques, we reveal notable increases in N_w_, ranging from 22 to 56%, during historical EWYs. These pulses are projected to amplify under the SSP126 (SSP370) scenario to 29 to 80% (61 to 120%) due to the projected increases in EWYs and higher nitrogen input. We identify the relative precipitation difference between two consecutive years (diffPr) as the primary driver of extreme N_w_. This finding forms the basis of the CLimate Extreme Adaptive Nitrogen Strategy (CLEANS), which scales down nitrogen input adaptively to diffPr, leading to a substantial reduction in extreme N_w_ with nearly zero yield penalty. Our results have important implications for global environmental sustainability and while safeguarding food security.

## Teaser

The CLEANS approach, which adapts N input to precipitation regime, shows potential to reduce N losses during extremely wet years.

## INTRODUCTION

Climate change is exacerbating extreme weather conditions at an alarming rate (*1*–*3*), as evidenced by extensive increases in the intensity, frequency, and duration of heavy precipitation events worldwide over the past five decades (*4*–*7*). These changes lead to more frequent extremely wet years (EWYs), which are characterized by strong positive anomalies of annual precipitation (*8*). EWYs pose widespread threats to various aspects of environmental and ecosystem sustainability (*9*, *10*). In particular, nitrogen (N) losses to aquatic ecosystems (N_w_) from agricultural land are substantially intensified during EWYs. For example, precipitation changes have led to up to a 33% increase in riverine N loads within the continental United States (*11*), causing severe freshwater eutrophication, groundwater contamination, and coastal phytoplankton blooms (*12*–*17*). Making things worse, a substantial portion of N_w_ eventually turns into N_2_O, a potent greenhouse gas, further exacerbating global warming (*18*). Unfortunately, EWYs are projected to increase further in the future (*19*). Thus, mitigating N_w_ and its associated pollution is a pressing priority in response to anthropogenic-accelerated climate extremes.

Previous studies have explored multiple strategies to mitigate N_w_ and address global N pollution, such as enhancing cropland N use efficiency, e.g., by reducing fertilizer application intensity (*20*) and optimizing other agricultural management practices (*21*–*24*). However, these studies have not adequately considered the impacts of EWYs. As the global population grows and becomes more affluent, food demand will increase. This will likely result in increased use of total N fertilizer, even with improved N use efficiency (*25*), which will make sustainable water quality management more challenging (*11*). In addition, the projected increases in the frequency and intensity of EWYs due to future climate change exacerbates the problem (*26*). The severity of the N problem ahead requires more effective strategies to limit N additions to control extreme N_w_ during EWYs (referred to as extreme N_w_ hereafter) without notably compromising crop yields.

We propose a CLimate Extreme Adaptive Nitrogen Strategy (CLEANS), which adapts N additions to EWYs. The rationale for CLEANS is inspired by long-term field experiments that reveal a substantial amount of residual N (>100 kg N ha^−1^) in soils during dry years (*27*), which could be used by crops in the following year, especially when it is a wet year, so as to reduce N additions without substantial impacts on crop yields. In principle, CLEANS is a practical and essential approach that needs urgent implementation in the face of climate change. Despite the potential benefits of CLEANS, its global significance, impact on extreme N_w_, and optimal application timing, crop targets, and locations are still not well understood. By addressing these questions, CLEANS has the potential to promote both environmental sustainability and food security, leading to a win-win outcome.

We aim to address these knowledge gaps by using the latest advancements in crop modeling [Python-based Environmental Policy Integrated Climate (PEPIC)] (*20*, *28*, *29*), bias-corrected general circulation models (GCMs), and machine learning [Random Forest (RF)], in combination with a comprehensive global dataset on crop yields, climate, management practices, and soil properties. We estimate the global magnitude of extreme N_w_ during both historical (1981–2010) and future (2036–2065) periods, assess its geospatial distribution according to food production units (FPUs) (*30*, *31*), and disentangle the effects of key factors, including management, climate, and soil-related variables. With this knowledge, we assess the CLEANS approach for mitigating extreme N_w_ with minimal compromises on crop yields. To illustrate the effectiveness of CLEANS under different climate scenarios from the Shared Socioeconomic Pathways and Representative Climate Pathways (SSP-RCPs), we focus on two contrasting trajectories: SSP126 and SSP370 (*32*).

## RESULTS

### Substantially higher N losses during EWYs

**Fig. 1.**
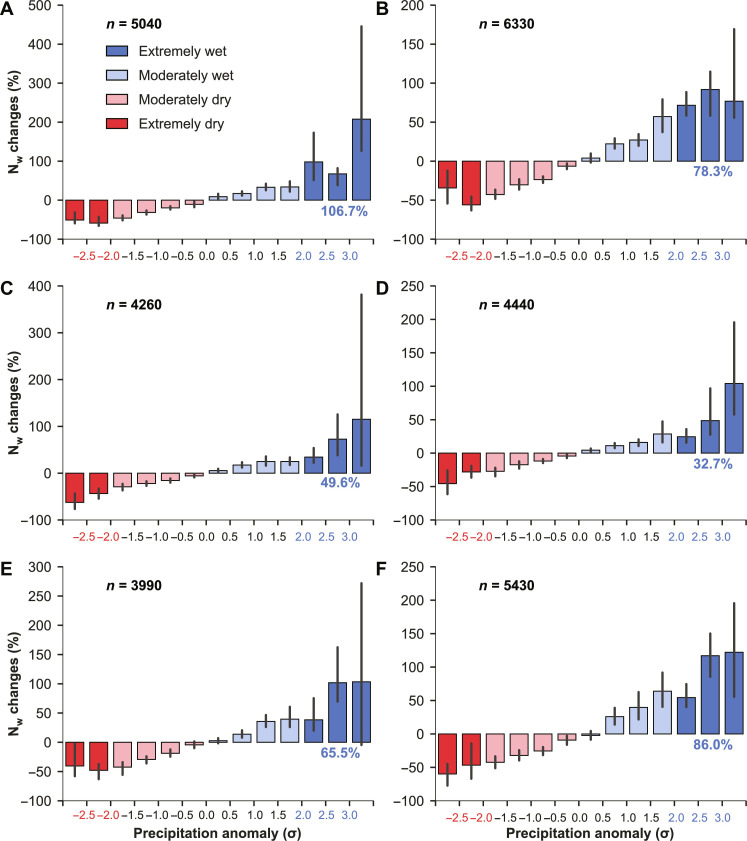
Relative changes in nitrogen losses (N_w_) under different precipitation anomalies. Bar plots show N_w_ changes relative to the 1981–2010 average for maize (**A** and **B**), rice (**C** and **D**), and wheat (**E** and **F**) presented separately for irrigation (left) and rainfed (right) conditions. Error bars represent 95% confidence intervals estimated from 1000 bootstrapped resampled sets. Numbers in blue indicate the average N_w_ changes with precipitation anomalies >2σ (i.e., extreme N_w_ changes). The number of FPU-year combinations is indicated by “*n*.”

During the historical period, EWYs with annual precipitation anomalies greater than 2σ (see Materials and Methods) occurred in less than 3% of all FPU-year combinations (fig. S1 and table S1). However, despite their rarity, EWYs caused significantly higher N_w_ than average, particularly for maize (Fig. 1). In terms of relative change, extreme N_w_ for maize exceeded the historical average by 78 and 107% under rainfed and irrigated cultivation, respectively. Furthermore, during years with annual precipitation anomalies greater than 3σ, extreme N_w_ was more than double the long-term averages for all crops, regardless of irrigation status. On the other hand, dry years showed lower N_w_ than the long-term averages, indicating that more N is likely stored in the soil during periods of low precipitation.

Notably, the magnitudes of extreme N_w_ varied considerably among global FPUs (fig. S2), with considerable effects observed in FPUs characterized by low average annual precipitation (fig. S3). In contrast, the patterns of N input and average N_w_ (figs. S4 and S5) were not associated with the magnitudes of extreme N_w_ across different FPUs. This result suggests that low extreme N_w_ is not necessarily linked to low N input and low average N_w_ losses. Although the number of EWYs was small (only 1 or 2 years of 30 in most cases; fig. S6), total N_w_ during these years accounted for more than 10 to 25% of the total N_w_ in most cases during the 1981–2010 period (fig. S7).

**Fig. 2.**
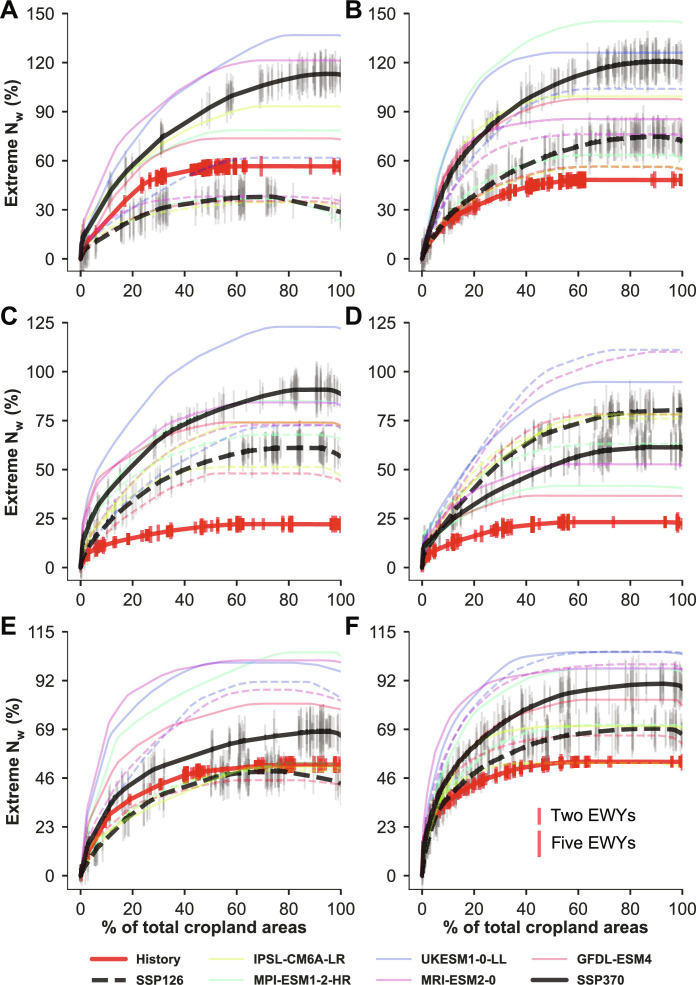
Cumulative extreme nitrogen losses (N_w_). Each line shows the cumulative extreme N_w_ relative to the 1981–2010 average along different FPUs. Extreme N_w_ is set to 0 for the regions without EWYs. Bold red lines indicate the extreme N_w_ during the historical period, while bold black lines present average extreme N_w_ among different climate models under future (2036–2065) SSP126 (dashed lines) and SSP370 (solid lines) conditions. Small vertical bars along the bold lines indicate the number of years falling in EWYs. Irrigated (left) and rainfed (right) conditions are distinguished for maize (**A** and **B**), rice (**C** and **D**), and wheat (**E** and **F**).

Globally, extreme N_w_ has a substantial impact on the deterioration of N pollution during both historical and future periods (Fig. 2). Cumulatively, extreme N_w_ across different FPUs was 22 to 56% higher than the historical average N_w_, depending on crop species and cultivation conditions. The area-weighted average for all three crops integrated from irrigated and rainfed cultivations was 43%. The extreme N_w_ are more severe in the future during EWYs and could reach up to 29 to 80% higher than of historical average N_w_, with an area-weighted average of 63% for the three crops under SSP126 and 61 to 120% (92% for the three crops) under SSP370. These numbers notably surpass the impact of historical EWYs. Over the historical period, ~60% of cropland was affected by EWYs, which is projected to increase to about 90%. However, there are differences in the future cumulative extreme N_w_ among different climate models, indicating uncertainty in predicting future EWY impacts. The projected increases in the EWY impacts are due to the combined effect of increases in precipitations over EWYs (fig. S8) and changes in future N input according to plausible social development scenarios (table S2). Although future precipitation (both long-term and during EWYs) under SSP370 is slightly lower than that under SSP126 during 2036–2065, increases of future extreme N_w_ are larger under SSP370 than under SSP126 due to higher future N input, except for rainfed rice. In addition, the frequency of the EWYs for future FPU years is projected to increase from less than 3% during the historical period to more than 20% (table S1 and fig. S9) using the mean and SD of historical precipitation to define future precipitation anomalies (see Materials and Methods).

**Fig. 3.**
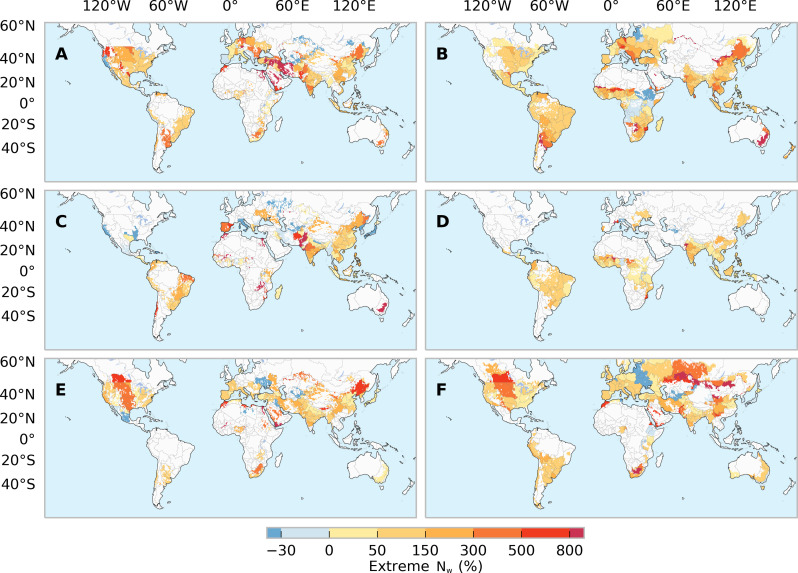
Changes in extreme nitrogen losses (N_w_) during 2036–2065 under the scenario SSP370. Maps show the differences (%) in extreme N_w_ averaged over five GCMs relative to the 1981–2010 average. Irrigated (left) and rainfed (right) conditions are distinguished for maize (**A** and **B**), rice (**C** and **D**), and wheat (**E** and **F**).

Spatially, future climate change is expected to increase extreme N_w_, and the areas affected by EWYs are projected to become more widespread (Fig. 3 and fig. S2). Specifically, compared to historical climate, extreme N_w_ under future SSP370 climate is estimated to increase by 500 to 800% for western Russia, Saudi Arabia, Iran, southeastern Australia, northeastern China, and central North America (Fig. 3). However, the severity of these increases is relatively lower under scenario SSP126 (fig. S10).

### Drivers of extreme N_w_

**Fig. 4.**
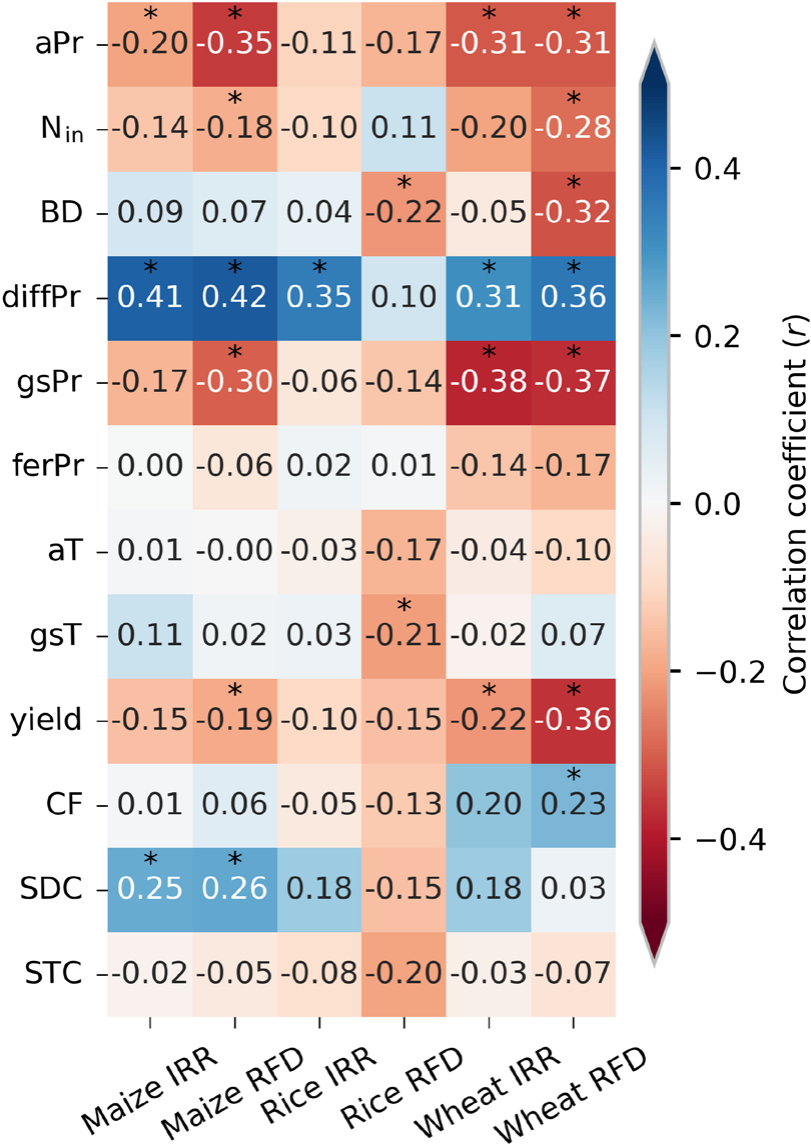
Correlation coefficient (r) between extreme nitrogen losses (N_w_) and different driving factors of the three crops under irrigated (*IRR*) and rainfed (*RFD*) conditions. The factors include annual precipitation (aPr), N input (N_in_), soil bulk density (BD), relative precipitation differences between two consecutive years (diffPr), growing season precipitation (gsPr), precipitation during N fertilization period (ferPr), annual temperature (aT), growing season temperature (gsT), crop yield, coarse fragment (CF), sand content (SDC), and silt content (STC). The asterisk indicates significance at 95%. Details of the correlation refer to figs. S11 to S13.

The most influential factors that drive extreme N_w_ are precipitation-related, specifically annual precipitation and the relative precipitation differences between two consecutive years (diffPr, see Materials and Methods) (Fig. 4 and figs S11 to S13). Unexpectedly, other factors such as N input, temperature, and soil properties have no consistent and statistically significant effects. The weak and negative relationship between N input and extreme N_w_ across FPUs suggests that reducing N input indiscriminately may not help decrease extreme N_w_. We found that extreme N_w_ tends to be higher during EWYs occurring over dry regions, indicating that lower precipitations are associated with higher extreme N_w_. For all crops, the variable diffPr has a strong positive impact on extreme N_w_ under both irrigated and rainfed conditions. This suggests that a substantial increase in precipitation in the current compared to the previous year could largely increase N pollution. Furthermore, diffPr has a significantly positive relationship with the precipitation anomaly of the current year but a significantly negative relationship with the precipitation anomaly of the previous year (fig. S14). The significant relationship between extreme N_w_ and diffPr could be explained by large N stored in soil (*27*) during previous dry conditions with less N_w_ (Fig. 1). As a result, high levels of N application in a current wet year, combined with large residual N from the previous year, exacerbate N_w_. We also found that there are differences in the spatial patterns between extreme N_w_ and the absolute changes in N_w_ during EWYs (fig. S15). Values of absolute changes in N_w_ during EWYs are stronger in wet regions with high N input. Therefore, reducing N fertilization during years characterized by high diffPr in regions with high N input could help reduce the risk of extreme N_w_.

### Mitigation of extreme N_w_

To identify efficient strategies to reduce extreme N_w_ without much impacting crop yields, we used machine learning models, specifically RF (*33*) models, to connect N_w_ and crop yield simulations from PEPIC with variables related to climate, management, and soil. The RF models were highly effective in predicting crop yields and N_w_ for the three crops, achieving an out-of-sample coefficient of determination (*R*^2^) greater than 0.92 and 0.85 for crop yields and N_w_, respectively (fig. S16). These RF models allow for greater flexibility and computational efficiency in assessing different options for mitigating extreme N_w_.

Scaling down N input every year across the historical period would decrease extreme N_w_ (fig. S17, A and B). However, this scenario would also lead to a notable reduction in crop yield over the same period, causing a substantial loss of crop production (fig. S17C). An alternative scenario is the CLEANS approach, which involves scaling down N input only in years characterized by high diffPr. By selecting various diffPr thresholds and scaling ratios of N input, the CLEANS method can have different effects on extreme N_w_, N input, and crop yield. When implemented optimally, it has the potential to substantially reduce extreme N_w_ with a moderate decrease in N input and only minor (or even no) effects on crop yield.

**Fig. 5.**
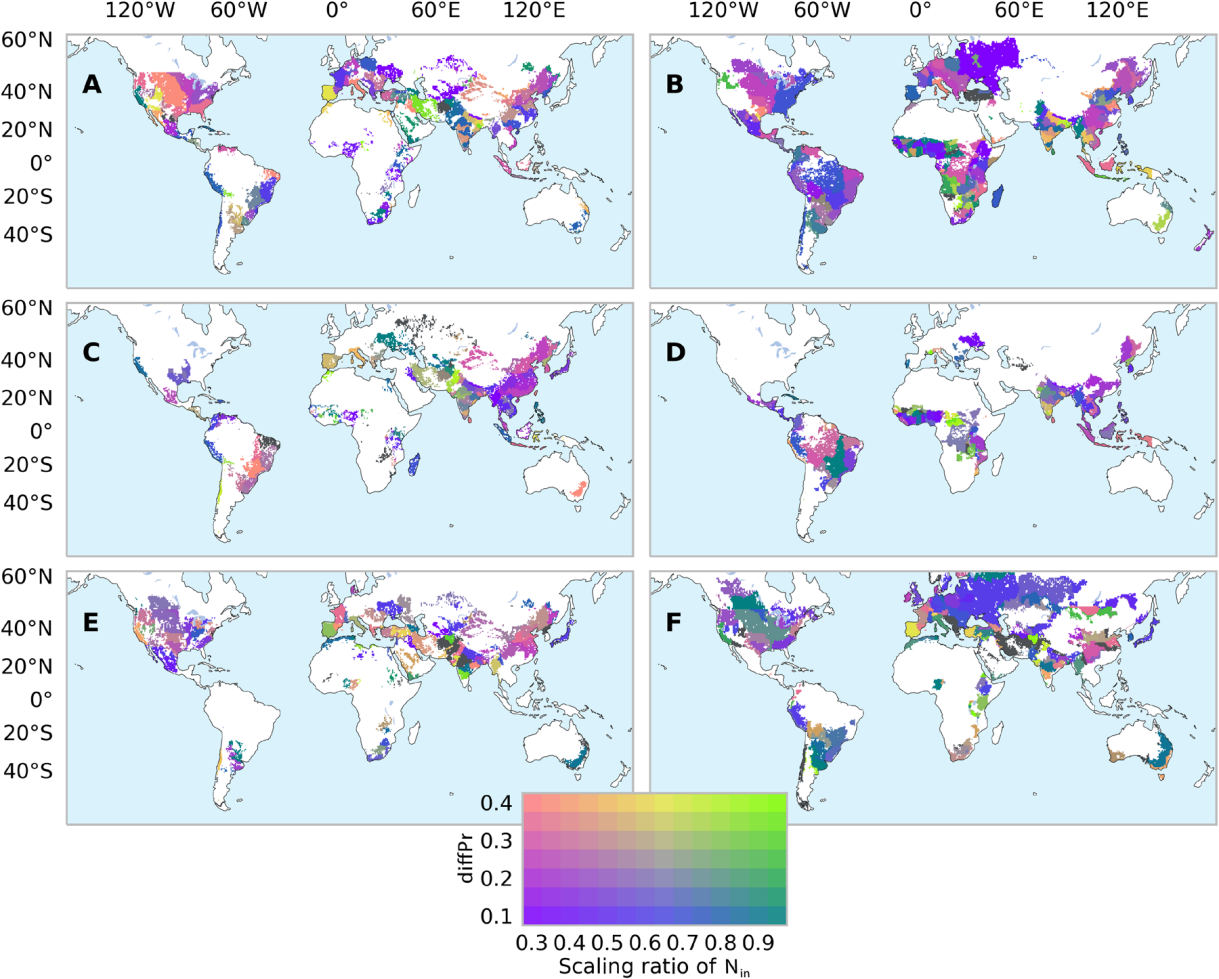
Optimal combinations of diffPr thresholds and scaling ratios of nitrogen input to reduce extreme nitrogen losses (N_w_). The maps illustrate, for each FPU, the best ratio for scaling down nitrogen input (N_in_) during years with a relative precipitation difference between two consecutive years (diffPr) higher than a certain threshold under irrigated (left) and rainfed (right) conditions for maize (**A** and **B**), rice (**C** and **D**), and wheat (**E** and **F**). These ratios result in the greatest reduction in extreme N_w_ while maintaining a <15% reduction in long-term N_in_ and a <3% reduction in crop yield under the SSP370 scenario. Regions shown in black indicate no optimal diffPr thresholds, and N_in_ scaling ratios were identified for the given reductions in N_in_ and yield constraints.

**Fig. 6.**
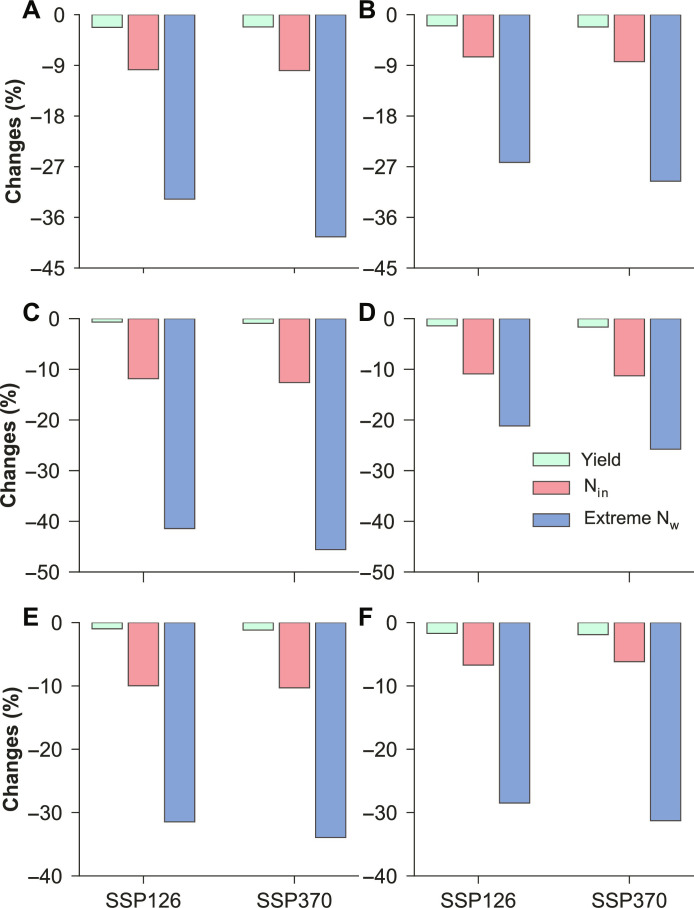
Mitigation of future extreme nitrogen losses (N_w_). The bars indicate changes in future extreme N_w_, average nitrogen input (N_in_), and crop yield for the period 2036–2065, with and without mitigation measures, relative to the 1981–2010 averages. The mitigation measures are achieved by using the diffPr thresholds and N_in_ scaling ratios shown in Fig. 5. Irrigated (left) and rainfed (right) conditions are distinguished for maize (**A** and **B**), rice (**C** and **D**), and wheat (**E** and **F**).

We demonstrated the effectiveness of CLEANS in reducing future extreme N_w_, under a scenario without any compromise on crop yield and a moderate reduction in long-term N input (<15%) for each FPU and crop. We identified the optimal diffPr thresholds and scaling ratios of future N input that allow the largest decreases in future extreme N_w_ in about 50% of FPUs without yield loss (fig. S18). However, with this no-yield loss strategy, reductions on future extreme N_w_ cannot be achieved everywhere, particularly for irrigated maize (fig. S19), because no optimal diffPr and scaling ratio was found in several major maize producing regions, e.g., the North China Plain. To achieve a high reduction in future extreme N_w_, we recommend implementing CLEANS with a slight crop yield loss, no more than −3%, and no more than −15% reduction on N input. In about 90% of FPUs, we detected the optimal diffPr thresholds and scaling ratios of future N input, which varied among FPUs and crops (Fig. 5 and fig. S20). In many regions, it is possible to scale down N input to a very low level (e.g., 0.3) and choose a small diffPr threshold (e.g., 0.1) (fig. S21). With these best threshold-scaling ratio combinations, future extreme N_w_ could potentially be decreased by 21 to 42% (26 to 46%) under the SSP126 (SSP370) scenario (Fig. 6). Future reductions in long-term crop yield are generally within 2% for both scenarios with a moderate reduction of N input by 10%. However, the exact changes in N_w_, N input, and crop yield vary with the management targets, as farmers and local governments may have varying levels of capacity to mitigate decreases in crop yield (e.g., −3 and −5%) and environmental needs to reduce N inputs (e.g., −10, −15, and −20%) (fig. S22). Despite these variations, the example provided above demonstrates the feasibility and substantial benefits of implementing CLEANS in reducing future extreme N_w_.

## DISCUSSION

This study sheds light on the disproportionate contribution of EWYs to past and future increases in N_w_, underscoring the important role of EWYs in driving N pollution. Our findings are consistent with previous studies (*34*, *35*) that identify N input as the primary factor controlling N_w_ (figs. S23 and S24), and N input is not the major driver of extreme N_w_ (*36*). Instead, the highest levels of extreme N_w_ tend to occur in regions with low precipitation but high variability in precipitation (diffPr) and are weakly associated with high N input. This is because regions with lower precipitation tend to have greater variability in precipitation (*37*), resulting in a wider range of extreme N_w_. Moreover, high diffPr values often reflect dry conditions in the preceding year, which can lead to the accumulation of N in the soil (*27*) and contribute substantially to N_w_ under subsequent wet conditions (*12*, *14*). This pattern was observed in the upper Mississippi basin, where the nitrate flux in 2013 was more than double the historical average due to the substantial residual N accumulated in soil during the 2012 drought (*38*). Therefore, our study highlights the need for future N mitigation strategies to account for the impact of EWYs and diffPr, as demonstrated by the CLEANS approach. These strategies will be critical to balancing food security and environmental risks.

Our CLEANS approach only aims to reduce N input during years with high diffPr values. This evidence-guided approach is most efficient when implemented adaptively, considering factors such as EWYs, crop types, local conditions, and specific management goals. To illustrate a feasible management target, we chose a crop yield reduction criterion of −3%, which could be compensated through innovative techniques and best management practices that boost crop yields (*39*, *40*). This flexible methodological framework can also accommodate different management targets. The divergent effects of crop type, climate, soil, and management-related variables on extreme N_w_ suggest that nonuniform mitigation efforts are necessary for reducing N_w_ during EWYs (*19*). The N problem is complex and dynamic, involving multiple biological, chemical, technical, societal, political, and economic dimensions. Thus, future implementations of CLEANS should be conducted in conjunction with advancements from other sectors. For example, some agricultural practices—such as enhanced-efficiency fertilizer, biochar additions, crop rotation, reduced tillage, and buffer zones—can lower N_w_, as highlighted in (*24*). In addition, government subsidies could provide an incentive for the implementation of CLEANS, especially among smallholders (*41*). Combining these practices with CLEANS could further reduce extreme N_w_ and benefit agriculture. However, local decision on fertilization practices is a rather complex issue, which is often currently determined by local feed and food production priorities rather than weather forecasts or climate projections (*35*, *42*, *43*). Nonetheless, we see the advantage in applying CLEANS for reducing N burdens in regions suffering from poor water quality.

The information derived from this study is crucial for ensuring sustainable agricultural development and other benefits for humanity and ecosystems (*23*, *44*). However, as with previous studies, uncertainties related to model simulations, GCMs, and future climate scenarios must be considered when implementing our CLEANS approach. It is particularly challenging to accurately predict future precipitation regimes, the frequencies of extremely wet conditions, and the diffPr values. Improved predictions of future EWYs could help mitigate these challenges (*45*–*47*). From a practical standpoint, CLEANS requires predicting diffPr before applying N fertilizer, which can be difficult for farmers. In these cases, governance, scientific communities, and agronomic practitioners should collaborate to transfer knowledge and provide relatively reliable middle-term weather forecasts. Although our study indicates that air temperature (both annual and growing season) is not among the main influencing factors of extreme N_w_ (Fig. 4 and fig. S12), it is an important driver of the recent intensification of global phytoplankton blooms (*17*, *48*). Therefore, air temperature needs to be considered, especially when dealing with the risk of eutrophication. Future research should investigate the effects of a concurrent occurrence of EWYs and warming conditions on eutrophication (*49*). Last, we did not distinguish the uptakes of different chemical forms of N, e.g., nitrate and ammonium. Some crops may have a preference for a specific form of N, e.g., rice preferring ammonium under certain conditions (*50*). These distinct preferences and different N uptake rates may affect the CLEANS approach, as dry conditions in 1 year could lead to the transformation of ammonium to nitrate and thus a lower ammonium level in the next (wet) year. However, crop preferences are uncertain and some studies also indicated that rice could take up more nitrate under acid conditions (*50*), and alternate wetting and drying conditions (*51*) making the magnitudes and impacts of the potential preferred N uptake difficult to quantify. The CLEANS approach may thus be in its generic form less robust in conditions with strong differences of uptake between ammonium and nitrate, and we suggest considering the preference of N when practically implementing the CLEANS approach. Despite our consideration of multifaceted uncertainties in the research outcomes, there are inherent uncertainties in our simulation framework. Differences among input data sources, especially in agricultural N input data (*39*, *52*, *53*), together with structural model uncertainty and approximations e.g., aggregation of various N forms and simplified representation of transformation processes in both terrestrial and aquatic environments (*54*), affect the results of our simulation. We, therefore, recommend interpreting the conclusions as outcomes derived from specific model and input data conditions. Future validation through observations at both field and regional scales for a comprehensive assessment could further strengthen the robustness of this study.

In summary, our research highlights the global importance of CLEANS, provides detailed and spatially explicit knowledge on its future implementation, and acknowledges that refinement of the approach requires collective efforts from scientists, farmers, and policy makers to codesign sound agriculture-environment-climate policy and practices in the future.

## MATERIALS AND METHODS

### Simulating aquatic nitrogen losses

To assess the impacts of EWYs, we used a large-scale crop model, PEPIC (*55*) to simulate major grain crops, including maize, rice, and wheat, along with their associated N dynamics on a global scale. The PEPIC model exhibited a good performance in simulating global N dynamics (fig. S25 and table S3) (*28*) and well represent crop yields across different countries (*29*) with *R*^2^ values ranging from 0.48 and 0.67 (fig. S26). Besides, we compared our simulated annual N_w_ of each FPU from the three crops to the observed total N concentration in the water bodies from the Global River Water Quality Archive dataset (*56*), both in normalized form, by estimating their correlation coefficients. Only the sites with observations of at least 10 years were chosen for the comparison. Given the simulated N_w_ is an aggregated value for each FPU, we then compared the simulated N_w_ with the site located at the most downstream region for each FPU. The results show that the correlation coefficients are higher than 0.5 in about two-thirds of the compared regions, especially in the United States and Canada, where longer observations are available (fig. S27). To estimate N_w_ and crop yields, we used the PEPIC model to simulate both irrigated and rainfed cultivations for each crop, with N input (from both chemical fertilizer and manure) applied twice during the growing season. The PEPIC model was then applied separately to simulate N_w_ for both historical (1981–2010) and future (2036–2065) periods at a spatial resolution of 30 arc min (about 50 km at the equator). Within PEPIC, N losses occur via volatilization and denitrification, as well as through surface runoff, leaching, and soil erosion, ultimately ending up in water bodies and the atmosphere. As the effects of EWYs on atmospheric N emissions are relatively minor (*57*) compared to the aquatic pathway, we focused on aquatic N losses in this study (*28*)

$$N_{w}=N_{\text{ws}}+N_{\text{wr}}+N_{\text{wl}}$$ (1)

where N_w_ is aquatic N losses and N_ws_, N_wr_, and N_wl_ are N losses to aquatic systems through soil erosion, surface runoff, and leaching, respectively. These indicators are in units of kg N ha^−1^ per year. To facilitate our analysis, we aggregated the gridded data into FPUs, which were defined based on river basins and economic regions introduced by Cai and Rosegrant (*30*) and modified by Kummu *et al.* (*31*), with a cropland area weighted method. We used a cropland area-weighted approach to ensure that the aggregated values were representative of the respective FPUs. We excluded FPUs with the smallest cropland areas, which accounted for a total of 0.05% of the global cropland of each crop.

### Defining EWYs

We applied the standardized anomaly (SA) method (*58*), which quantifies the deviations of annual precipitation from the multiyear average to quantify the different precipitation intervals in different FPUs. The SA of annual precipitation for each FPU during the historical period was calculated as

$${\text{SA}}_{i,t}=\frac{{\text{aPr}}_{i,t}-\bar{{\text{aPr}}_{i}}}{\sigma_{i}}$$ (2)

where SA*_i,t_* refers to the SA of precipitation in year *t* for FPU *i*, aPr*_i,t_* is annual precipitation in year *t* for FPU *i*, $\bar{{\text{aPr}}_{i}}$ means multiyear average precipitation of FPU *i*, and σ*_i_* is the SD of annual precipitation for FPU *i*. For the entire period (1981–2010), precipitation intervals were set as −2.5σ to +3.5σ, at an interval of 0.5σ, following Li *et al. * (*58*). A year was classified as an EWY if its SA value was greater than 2σ. For each FPU, the average N_w_ was estimated for the years in each precipitation anomaly interval. The average relative N_w_ change (in %) for a given precipitation anomaly interval from the average N_w_ over the entire period (30 years here) was used to determine the general impacts of precipitation anomalies on N_w_ considering all FPUs. We paid particular attention to the relative N_w_ change during EWYs, which we referred to as extreme N_w_ in this study. Because of high uncertainties in N_w_ simulations (*28*), we used 1000 times bootstrap to detect the uncertainties of extreme N_w_. We also estimated future precipitation anomalies (2036–2065) with Eq. 2 but using historical $\bar{\text{aPr}}$ and σ to show the future precipitation changes relative to historical climate condition.

### Cumulative extreme N_w_ and the influencing drivers

To illustrate the impact of EWYs on N_w_, we plotted the cumulative extreme N_w_ across FPUs in ascending order based on the difference between the average N_w_ during EWYs and the average N_w_ during the entire historical period. We also cumulated the future extreme N_w_ relative to the historical long-term average N_w_ to assess the severity of future extreme N_w_.

Our analysis considered various factors, including climate, management, and soil-related variables, to identify the major influencing factors of extreme N_w_. Climate variables such as annual precipitation (aPr), growing season precipitation (gsPr), precipitation during the period of two fertilization applications (ferPr), annual air temperature (aT), and growing season air temperature (gsT) were taken into account. In addition, the precipitation difference between two consecutive years relative to the multiyear average (diffPr) was also considered in the regression analysis to represent the possible cumulative effects on N_w_ due to dry conditions in the previous year

$${\text{diffPr}}_{i,t}=\frac{{\text{aPr}}_{i,t}-{\text{aPr}}_{i,t-1}}{\bar{{\text{aPr}}_{i}}}$$ (3)

where aPr_*i,t*−1_ is annual precipitation in year *t-1* for FPU *i*. Management factors consider N input, as well as irrigation/rainfed condition. Soil properties include bulk density (BD), coarse fragment (CF), sand content (SDC), and silt content (STC). Linear regressions between extreme N_w_ and the influencing drivers across FPUs were used to determine the major drivers.

### RF simulations

We used the RF (*33*) machine learning models, implemented through the ranger() function in the R programming environment, to train the N_w_ data simulated with PEPIC. To account for potential disparities in the response of N_w_ to climate variations, soil textures, and other influencing variables (e.g., N_in_), a specific RF model was built for each of the three crops at the FPU level. The model inputs included those considered in the regression analysis (as mentioned earlier). In addition, we introduced an independent factor variable “IRRF” (1 represents irrigated and 0 rainfed) in the model to distinguish between irrigated and rainfed cultivations. To distinguish between extremely and non-EWYs, we introduced another independent factor variable “Extreme” (1 represents EWYs and 0 other years) based on the SA value in each year. Given the strong correlation between aPr and gsPr, as well as between aT and gsT (as revealed by heat maps of autocorrelation in fig. S28), gsPr and gsT were excluded in the RF model, considering that aPr and aT were more important in representing N_w_.

To build the RF models, we divided the dataset into two parts, with 80% as the training dataset and 20% as the testing dataset. We removed outliers in both datasets using a chi-square test (*59*). The RF models were trained and optimized using 10-fold cross-validation to determine the best hyperparameters, including “mtry” and “ntree.” In each random forest tree, a random subset of the training dataset was used to learn from a random number of features (mtry) to split the tree. Predictions were made by averaging the predictions of all decision trees. We compared the PEPIC-simulated N_w_ with RF predictions and evaluated the performance of the RF models by calculating the root mean square error (RMSE) after each cycle of 10-fold cross-validation. The final hyperparameter values were chosen to minimize RMSE according to the cross-validation conducted with the training set. We tested the final model performance with the testing dataset and found that the *R*^2^ was higher than 0.85 (fig. S16). In addition, we built an RF model to analyze PEPIC-simulated crop yields using a similar approach, obtaining satisfactory predictive performance with an *R*^2^ higher than 0.92 based on the testing dataset (fig. S16). To analyze the relationship between N_w_ and each input variable, we used one-dimensional partial dependence plots (*60*) by averaging over all other predictors.

### The CLEANS approach

The RF models were used to investigate the potential mitigation of extreme N_w_ while minimizing the impact on crop yield. Regression analyses were conducted to identify the influential factors driving the increase in extreme N_w_, with diffPr found to be the most important factor (Fig. 4). Subsequently, the CLEANS approach was proposed to attenuate extreme N_w_. This approach involves scaling down N input to a specified ratio only for years with diffPr above a certain threshold. The CLEANS approach was applied to both historical and future periods, with varying diffPr thresholds and N input scaling ratios resulting in different effects on extreme N_w_ and crop yield. Combinations of diffPr thresholds and N input scaling ratios were selected for each region and crop to maximize the decrease in extreme N_w_ with constraining reduction of crop yield (<3%) and N input (<15%). The optimal combinations were determined by analyzing the highest reduction in extreme N_w_. To account for uncertainties, different constraints were set on reducing crop yield (e.g., 3 and 5%) and N input (10, 15, and 20%).

### Datasets

Climate data for the historical PEPIC simulation were obtained from the AgMERRA dataset (*61*) (available at http://data.giss.nasa.gov/impacts/agmipcf/agmerra), which provides daily air temperature, precipitation, solar radiation, relative humidity, and wind speed. The AgMERRA dataset has good representation of extreme climate and was mainly designed for detecting the impacts of extreme climate on crop production (*61*). Future (2036–2065) bias-corrected climate data were obtained from the Inter-Sectoral Impact Model Intercomparison Project (ISIMIP; available at www.isimip.org/) data archive, under two SSP-RCP scenarios (SSP126 and SSP370) from the Coupled Model Intercomparison Project phase 6 using five GCMs: MRI-ESM2-0, MPI-ESM1-2-HR, UKESM1-0-LL, GFDL-ESM4, and IPSL-CM6A-LR. Land cover data were derived from the SPAM2010 dataset (*62*), while crop calendar was obtained from the SAGE dataset (*63*). Historical cropland use and crop calendar information were also used for the future period to isolate the effects of climate change on N losses. Historical N input data for each crop, including mineral fertilizers and manure, were obtained from the EarthStat datasets (available at www.earthstat.org). The EarthStat datasets were derived from (*39*, *64*). Mineral N input was compiled from multiple data sources, including national and subnational sources, while the manure N input was derived from livestock density and mapped proportionally to cropland and pasture. The EarthStat N input data have been widely used in global studies (*42*, *65*–*67*) and provide crop-specific N input. Deposited N was estimated by the product of N concentration in rainfall and rainfall volume (*54*). Considering future changes in N input could be an important influencing factor of extreme N_w_, we used Land-Use Harmonization (LUH2) data (*68*) (available at https://luh.umd.edu) for years 2036–2065, similar to (*69*) This dataset contains both C3 annual crops (such as wheat and rice) and C4 annual crops (such as maize). Soil data—including the depth of each soil layer, BD, SDC, and STC—were obtained from the ISRIC–World Soil Information World Inventory of Soil Emission Potentials (ISRIC-WISE) dataset (*70*).

## Supplementary Materials

### This PDF file includes:

Figs. S1 to S28 Tables S1 to S3 References
