# Supplementary material for: Mitigating nitrogen losses with almost no crop yield penalty during extremely wet years
Source: Sci Adv. 2024 Feb 28;10(9):eadi9325. doi: 10.1126/sciadv.adi9325 (PMC10901370; doi:10.1126/sciadv.adi9325)
Supplement: Supplementary file 1 — Figs. S1 to S28 Tables S1 to S3 References [file sciadv.adi9325_sm.pdf]

Science Advances
AAAS

Supplementary Materials for

Mitigating nitrogen losses with almost no crop yield penalty during extremely wet years

Wenfeng Liu *et al.*

Corresponding author: Wenfeng Liu, wenfeng.liu@cau.edu.cn, wenfengliu@vip.sina.com

Sci. Adv. **10**, eadi9325 (2024)
DOI: 10.1126/sciadv.adi9325

This PDF file includes:

Figs. S1 to S28
Tables S1 to S3
References

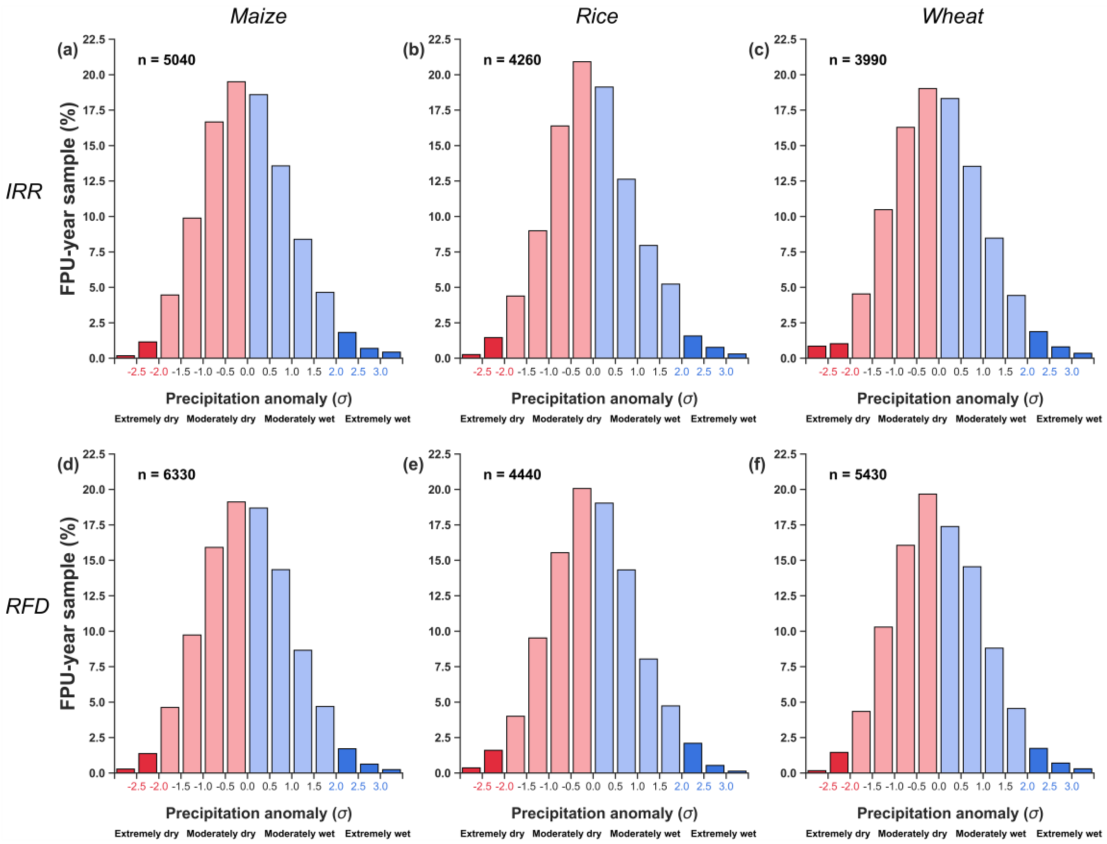

Fig. S1. Ratio of food production unit (FPU)-year combinations in each precipitation anomaly interval to all FPU-year combinations along the precipitation anomalies. Blue bars (precipitation anomaly > 2σ) represent the extremely wet intervals, while red bars the extremely dry intervals. Irrigated (IRR) and rainfed (RFD) are distinguished for three crops: maize (a, d), rice (b, e), and wheat (c, f).

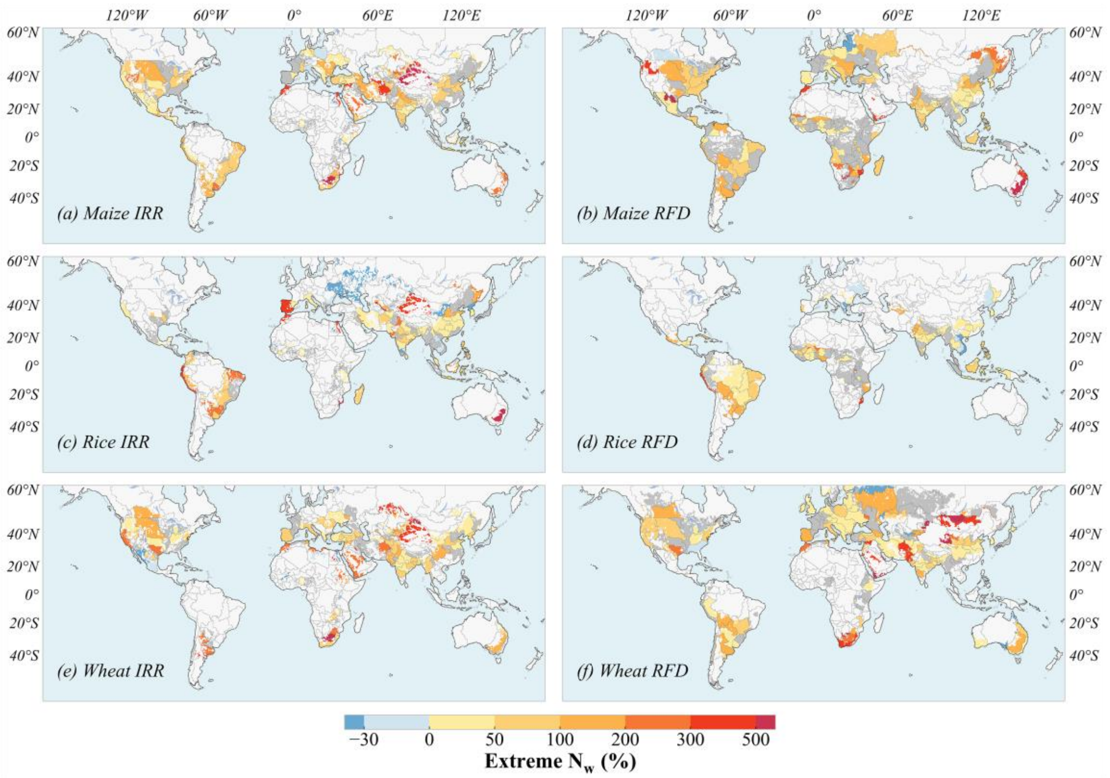

Fig. S2. Extreme aquatic nitrogen loss (N_w) anomalies during the historical period. Silver areas represent the food production units with no extremely wet years and white areas represent no cropland areas. Irrigated (IRR) and rainfed (RFD) are distinguished for three crops: maize (a, b), rice (c, d), and wheat (e, f).

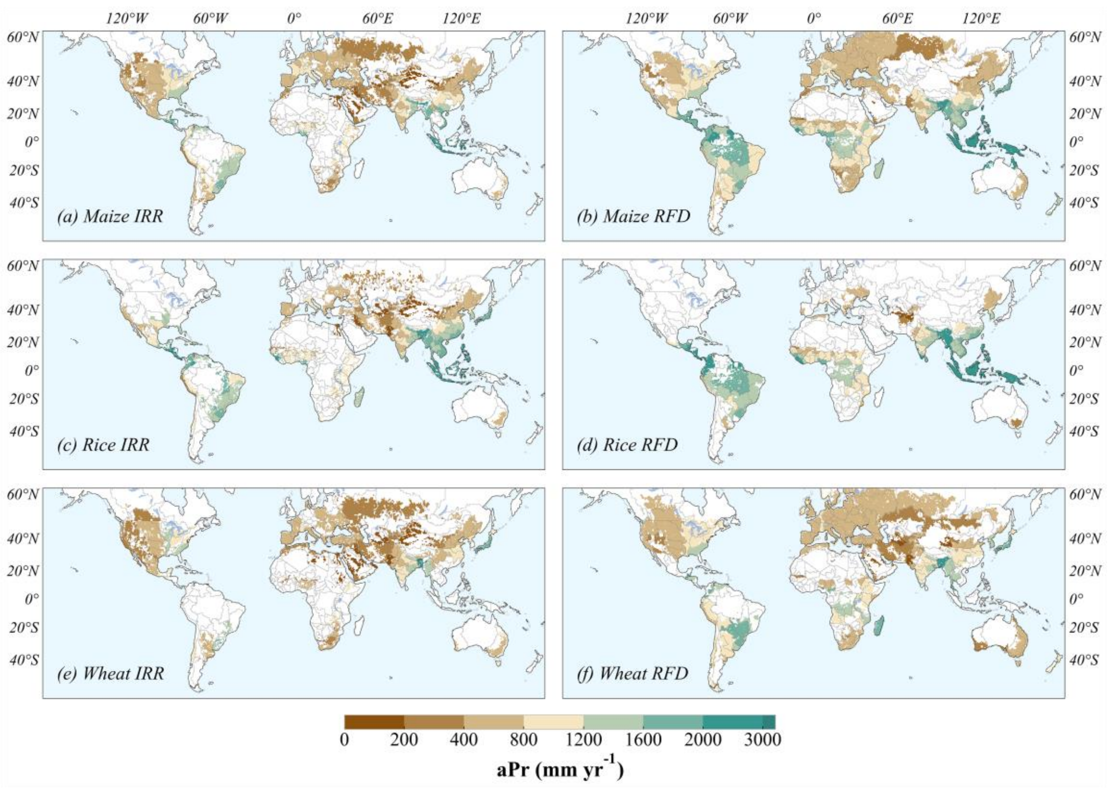

Fig. S3. Average annual precipitation (aPr) over the period 1981–2010. Irrigated (IRR) and rainfed (RFD) are distinguished for three crops: maize (a, b), rice (c, d), and wheat (e, f).

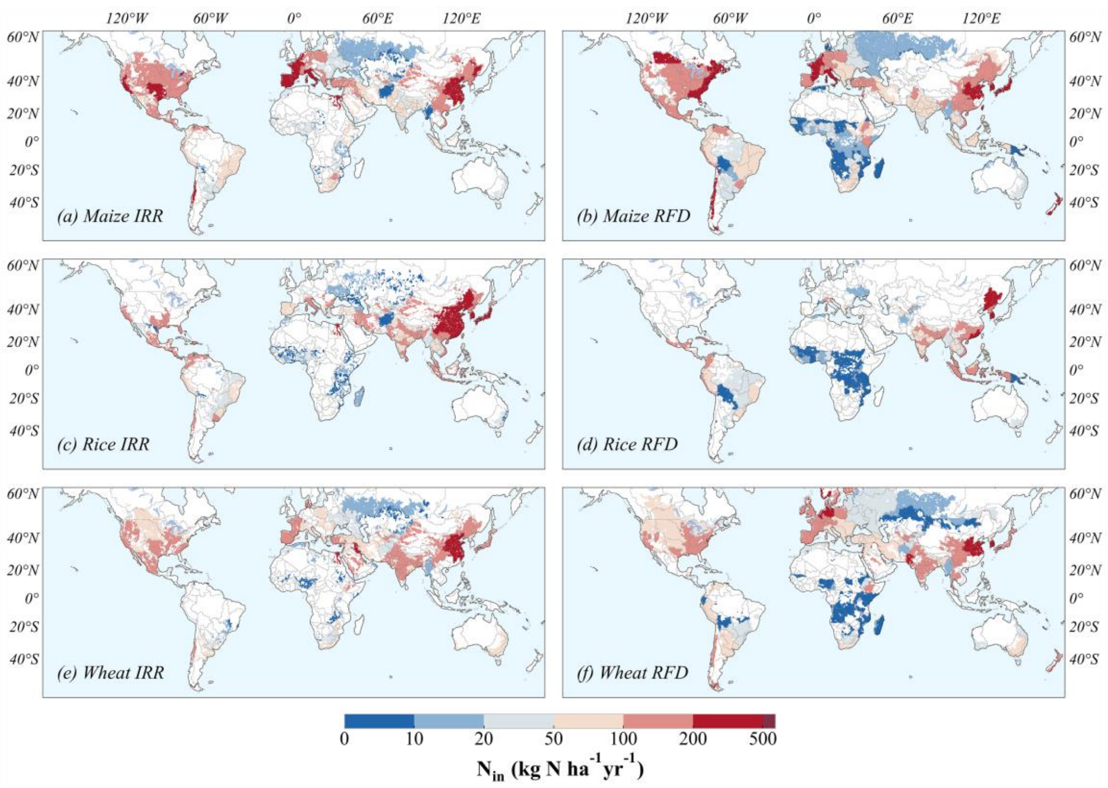

Fig. S4. Historical nitrogen input (N_{in}) based on the EarthStat dataset (*39, 64*). Irrigated (IRR) and rainfed (RFD) are distinguished for three crops: maize (a, b), rice (c, d), and wheat (e, f).

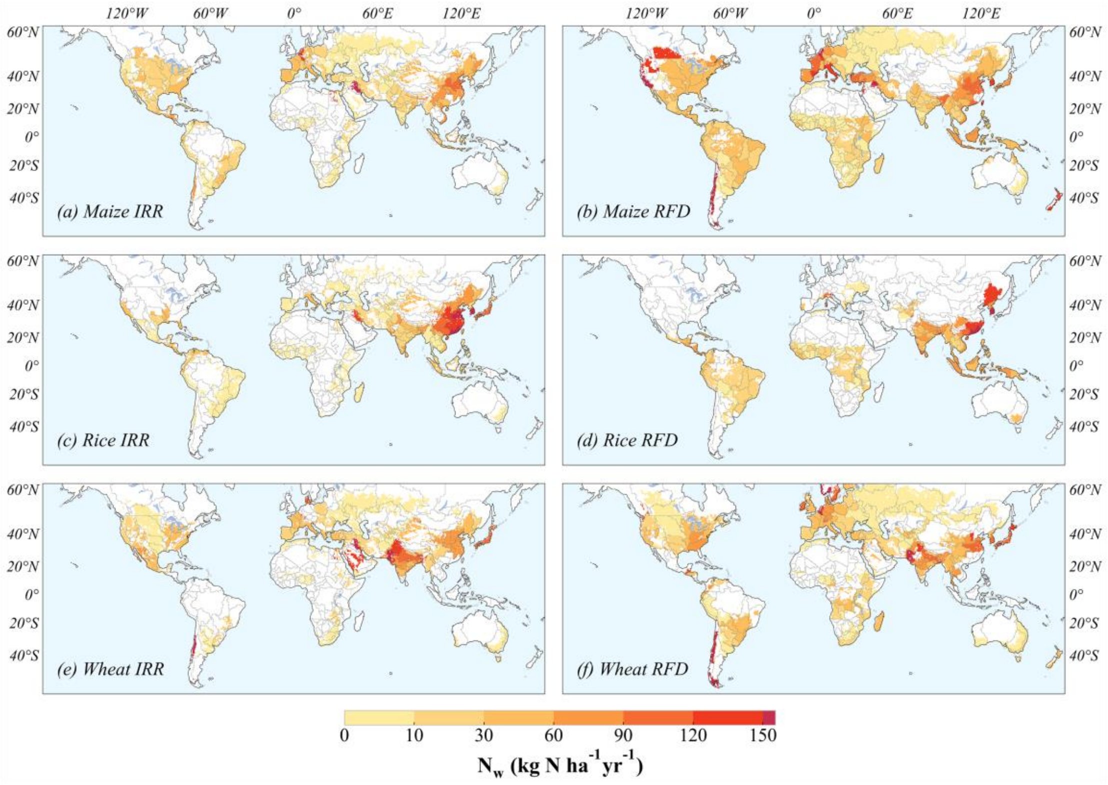

Fig. S5. Average aquatic nitrogen losses (N_W) over the historical period 1981–2010. Irrigated (IRR) and rainfed (RFD) are distinguished for three crops: maize (a, b), rice (c, d), and wheat (e, f).

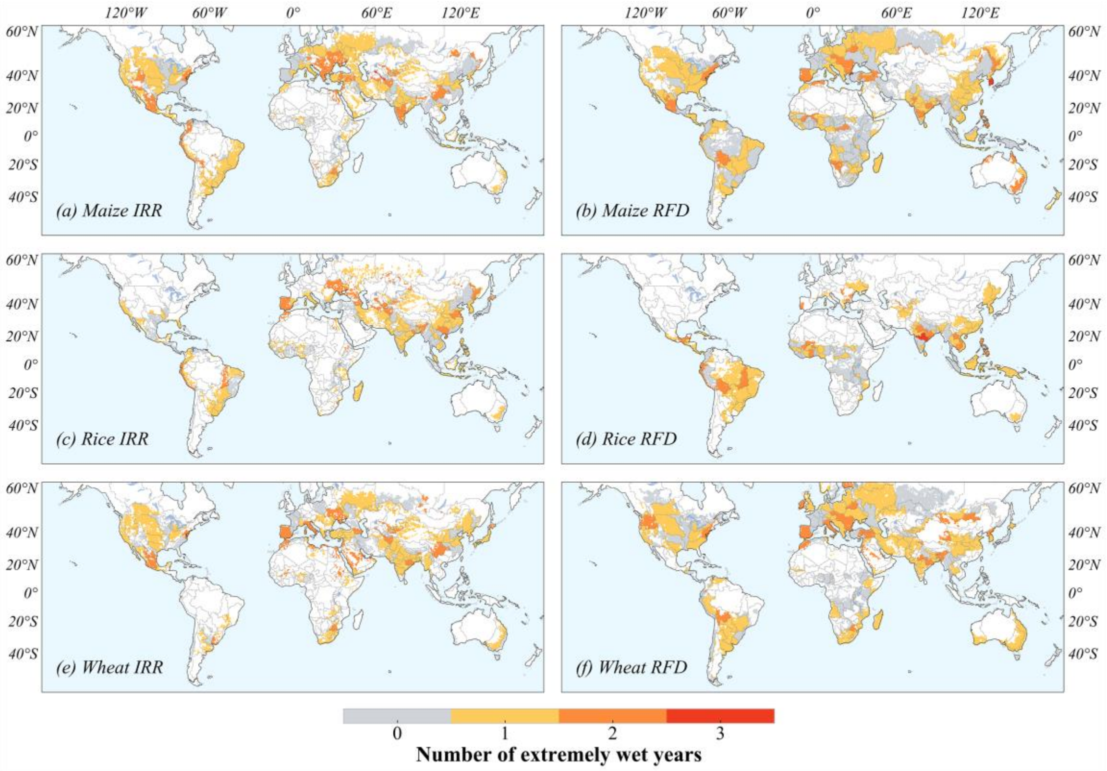

Fig. S6. Number of extremely wet years over the historical period 1981–2010. Irrigated (IRR) and rainfed (RFD) are distinguished for three crops: maize (a, b), rice (c, d), and wheat (e, f).

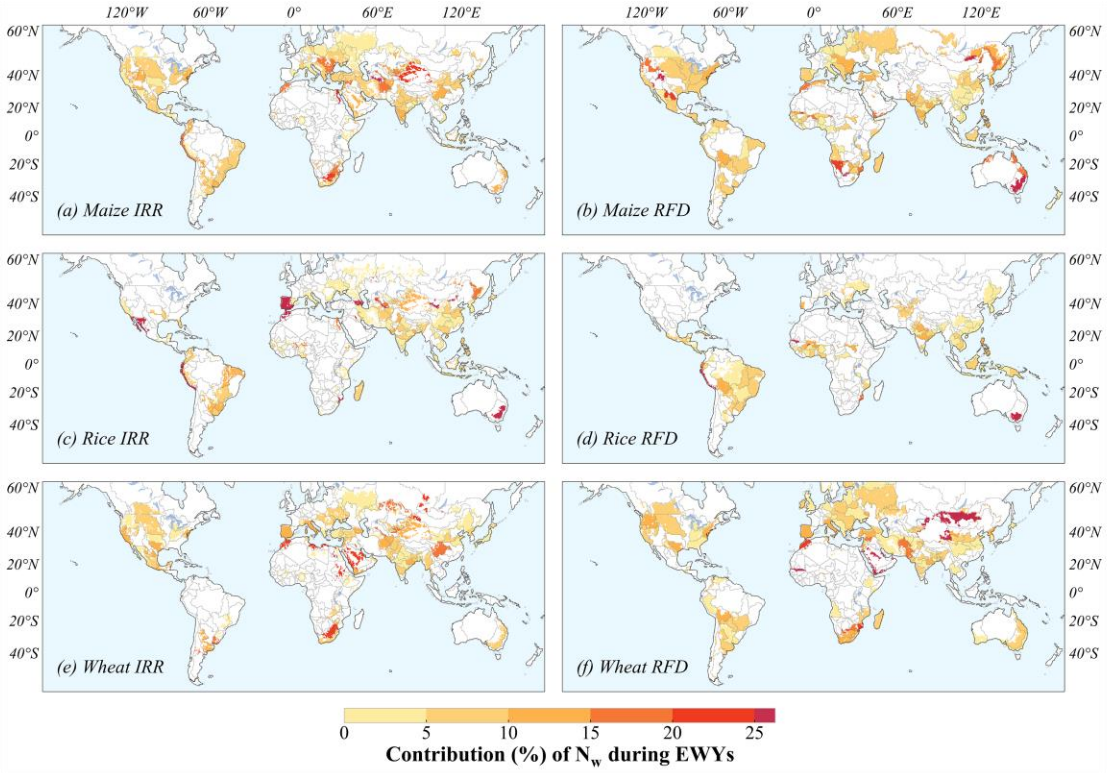

Fig. S7. Contribution of aquatic nitrogen losses (N_w) during extremely wet years (EWYs) to total N_w over the entire 1981–2010 period. Irrigated (IRR) and rainfed (RFD) are distinguished for three crops: maize (a, b), rice (c, d), and wheat (e, f).

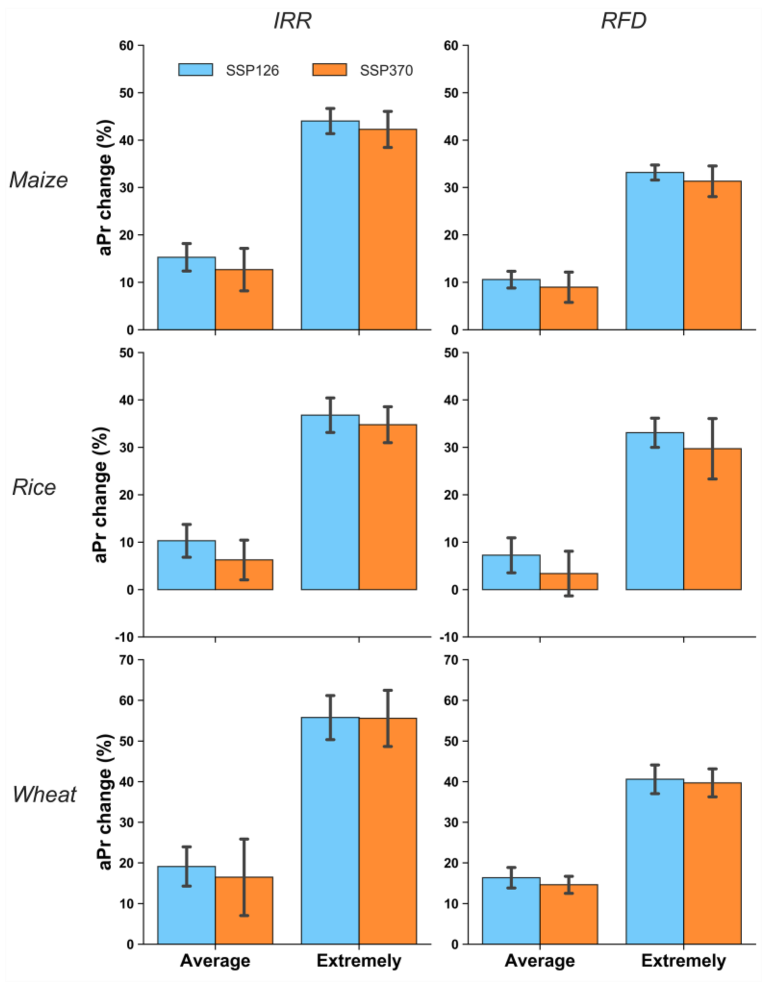

Fig. S8. Changes in future annual precipitation (aPr) relative to 1981–2010 average. ‘Average’ means changes in aPr average over future 30 years, weighted by food production unit (FPU) cropland areas, relative to the 1981–2010 average; ‘Extremely’ means changes in aPr average over future extremely wet years relative to the 1981–2010 average. IRR: irrigated; RFD: rainfed.

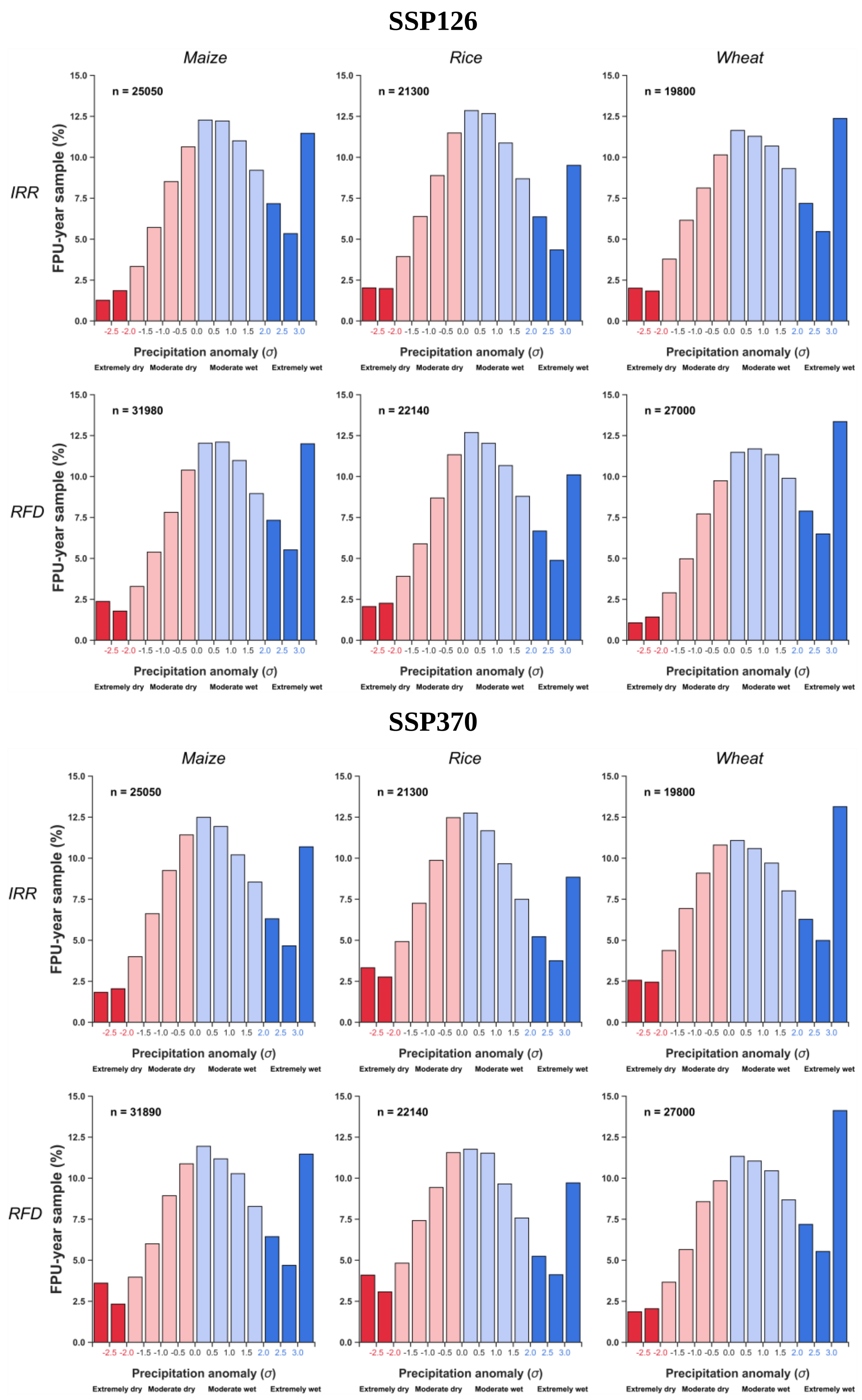

Fig. S9. Similar to fig. S1 but for the 2036–2065 period based on the SSP126 (upper panel) and SSP370 (lower panel) scenarios. The precipitation distribution presents the overall samples distribution of the five climate models. Each panel is

organized with crops (maize, rice, wheat) as columns, and distinguished between irrigated (IRR) and rainfed (RFD) conditions.

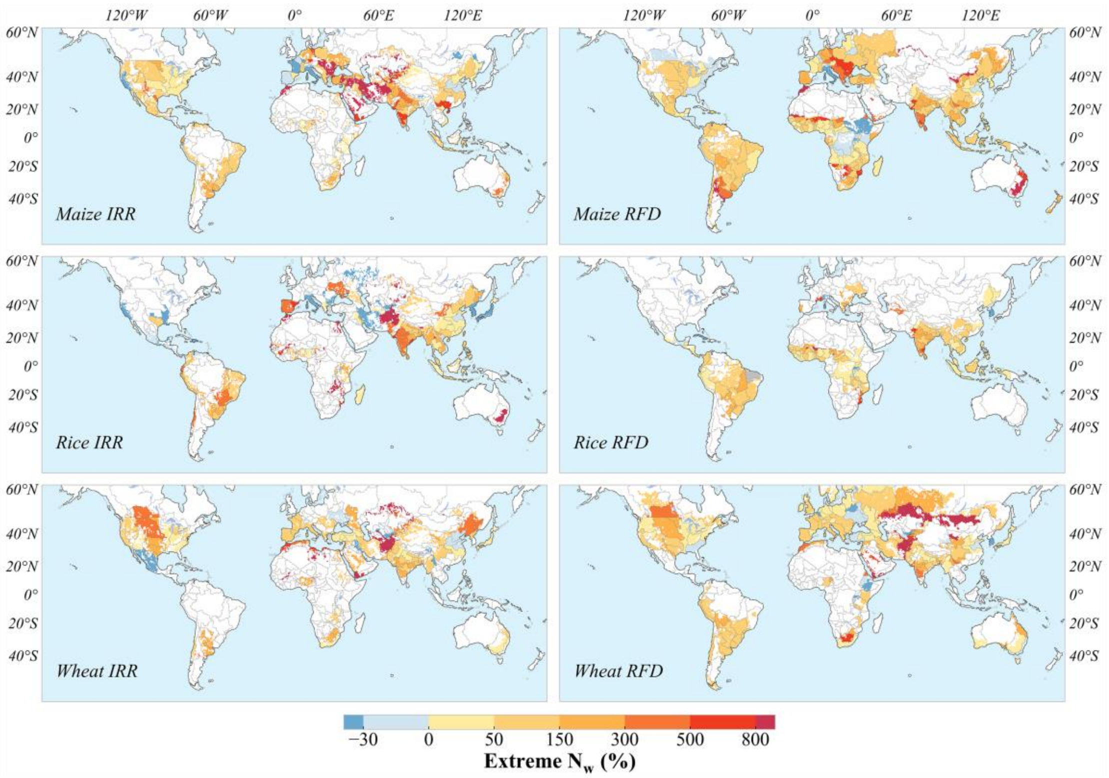

Fig. S10. Similar to Fig. 3 in the main text, but based on the scenario SSP126. Irrigated (IRR) and rainfed (RFD) are distinguished for three crops: maize, rice, and wheat.

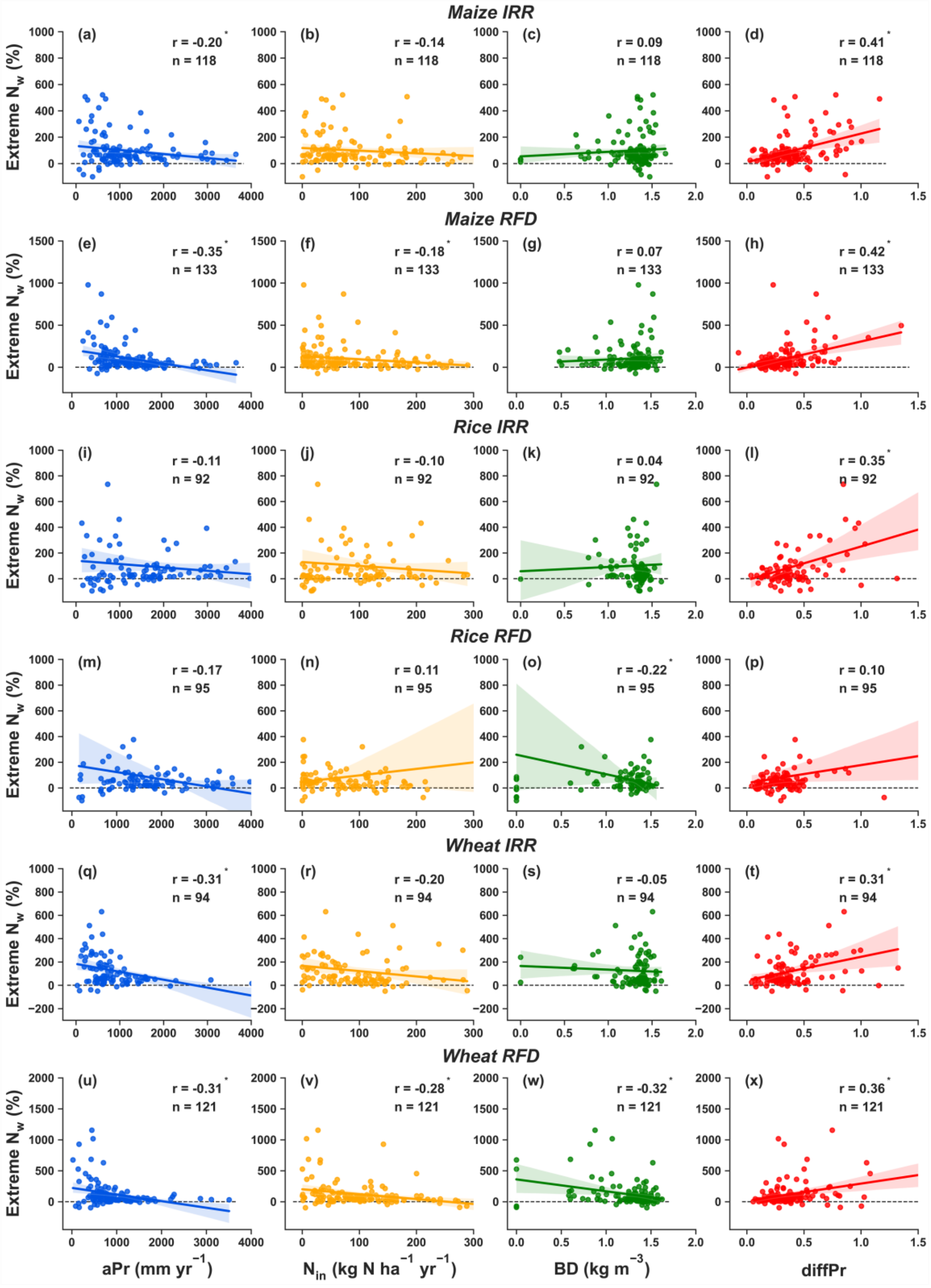

Fig. S11. Correlation between extreme nitrogen losses (N_w) and driving factors.

Each point represents the extreme N_w of a food production unit against various factors averaged over historical extremely wet years. The solid lines indicate the best-fit linear regressions between extreme N_w and driving factors, while the shaded areas

represent the 95% confidence intervals of 1,000 bootstraps. The “r” is the correlation coefficient, with an asterisk indicating significance at 95%. The factors include annual precipitation (aPr), N input (N_{in}), soil bulk density (BD), and relative precipitation differences between two consecutive years (diffPr). Irrigated (IRR) and rainfed (RFD) conditions are distinguished for maize (a-h), rice (i-p), and wheat (q-x).

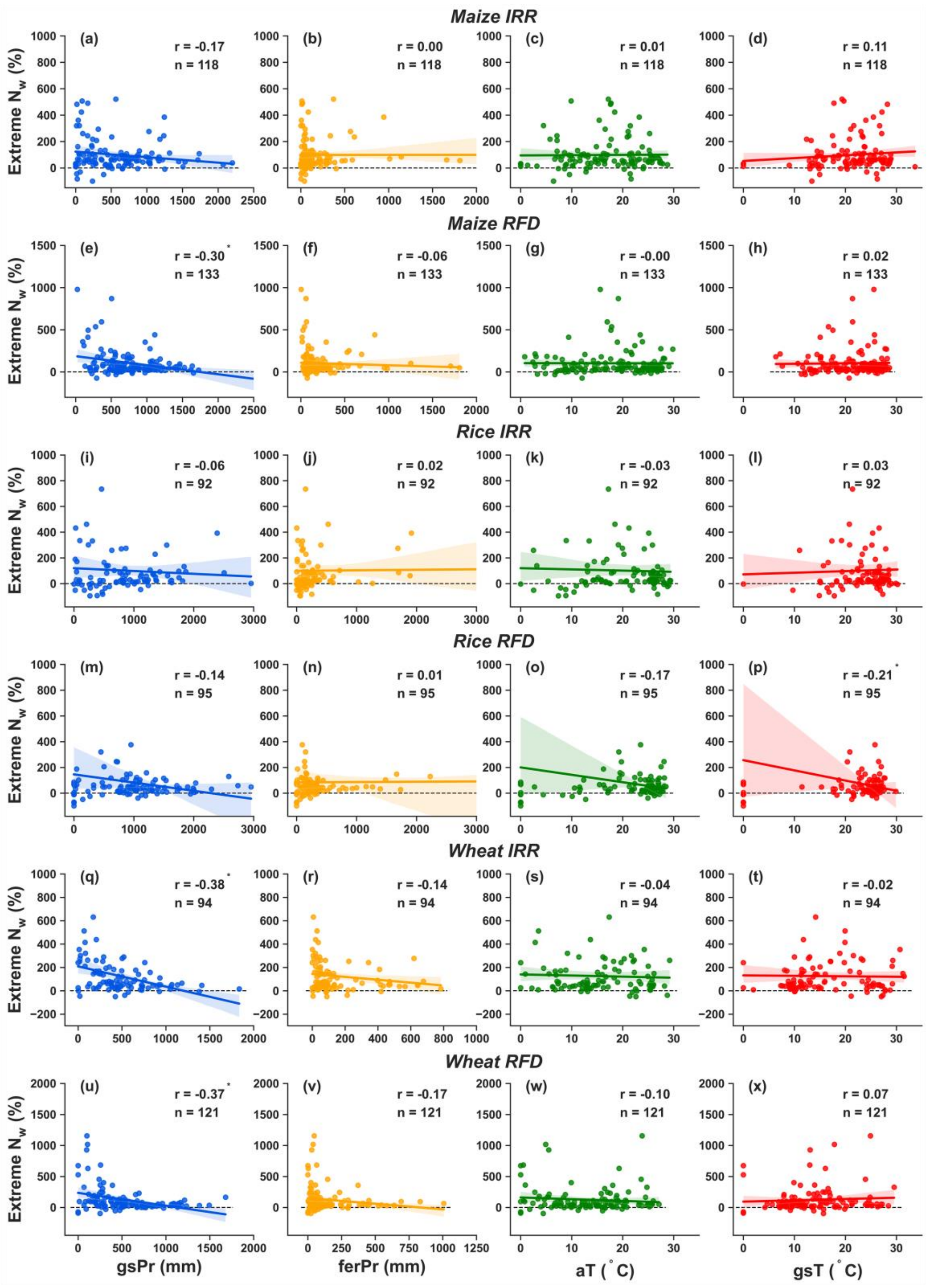

Fig. S12. Similar to fig. S11, but the influential drivers are growing season precipitation (gsPr), precipitation during N fertilization period (ferPr), annual temperature (aT), and growing season temperature (gsT). Irrigated (IRR) and rainfed (RFD) conditions are distinguished for maize (a-h), rice (i-p), and wheat (q-x).

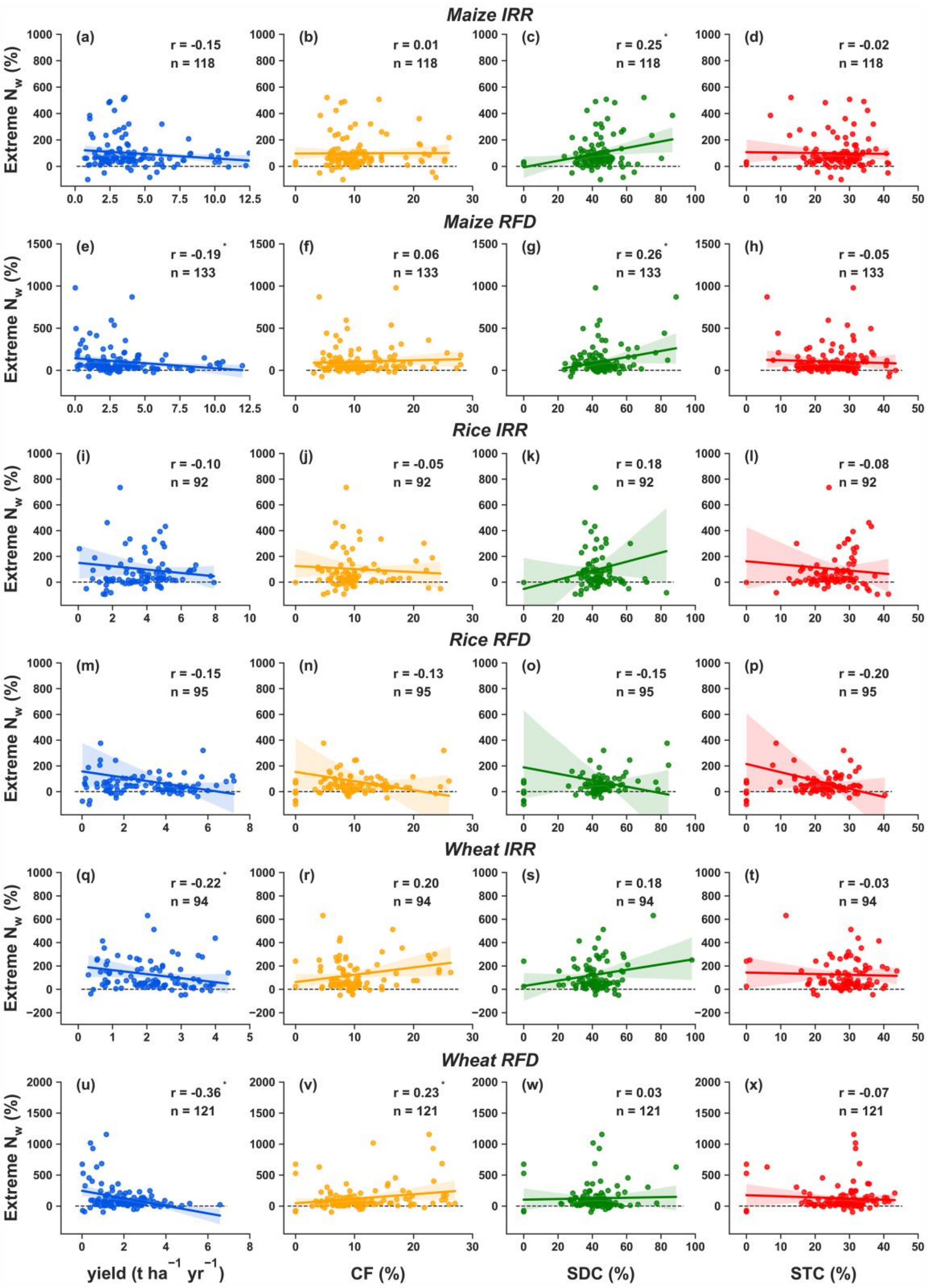

Fig. S13. Similar to fig. S11, but the influential drivers are crop yield, coarse fragment (CF), sand content (SDC), and silt content (STC). Irrigated (IRR) and rainfed (RFD) conditions are distinguished for maize (a-h), rice (i-p), and wheat (q-x).

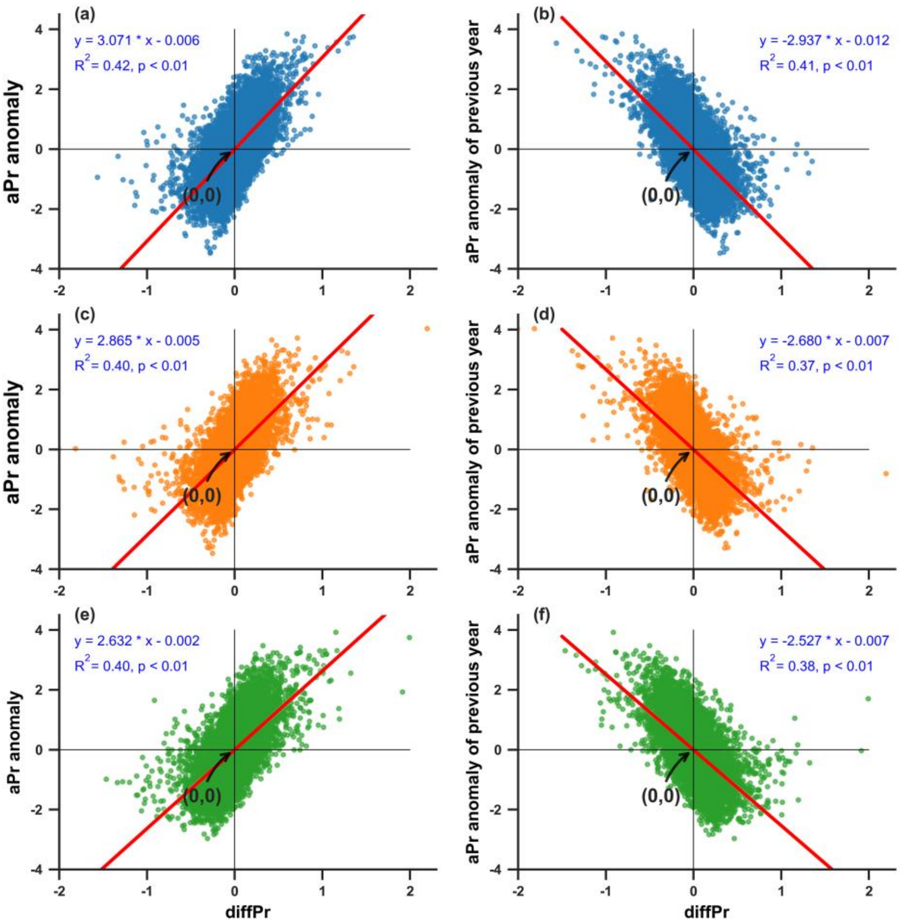

Fig. S14. Relations between annual precipitation (aPr) anomaly and relative precipitation difference between two consecutive years (diffPr). Equations show the linear regression between aPr and diffPr with coefficient of determination (R^2) and p-value presented. Red lines are the linear regression lines. The relations are presented for three crops: maize (a, b), rice (c, d), and wheat (e, f).

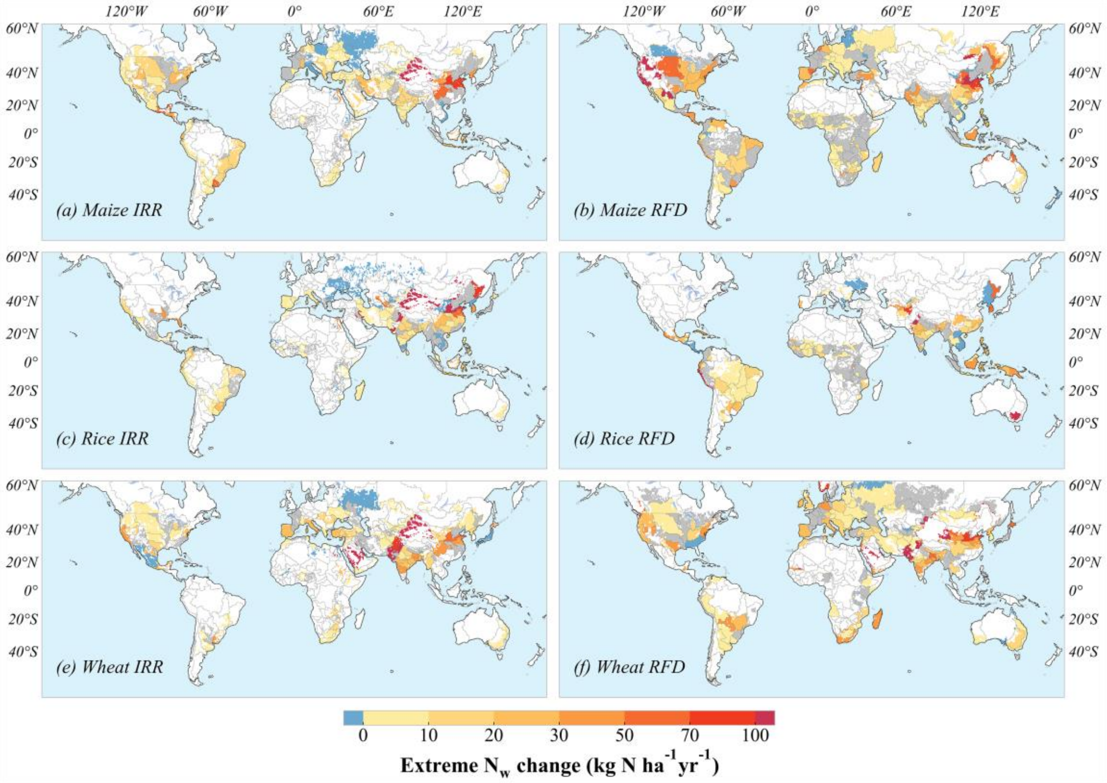

Fig. S15. Absolute extreme aquatic nitrogen loss (N_W) changes during the historical period. Silver areas represent the food production units with no extremely wet years and white areas represent no cropland areas. Irrigated (IRR) and rainfed (RFD) are distinguished for three crops: maize (a, b), rice (c, d), and wheat (e, f).

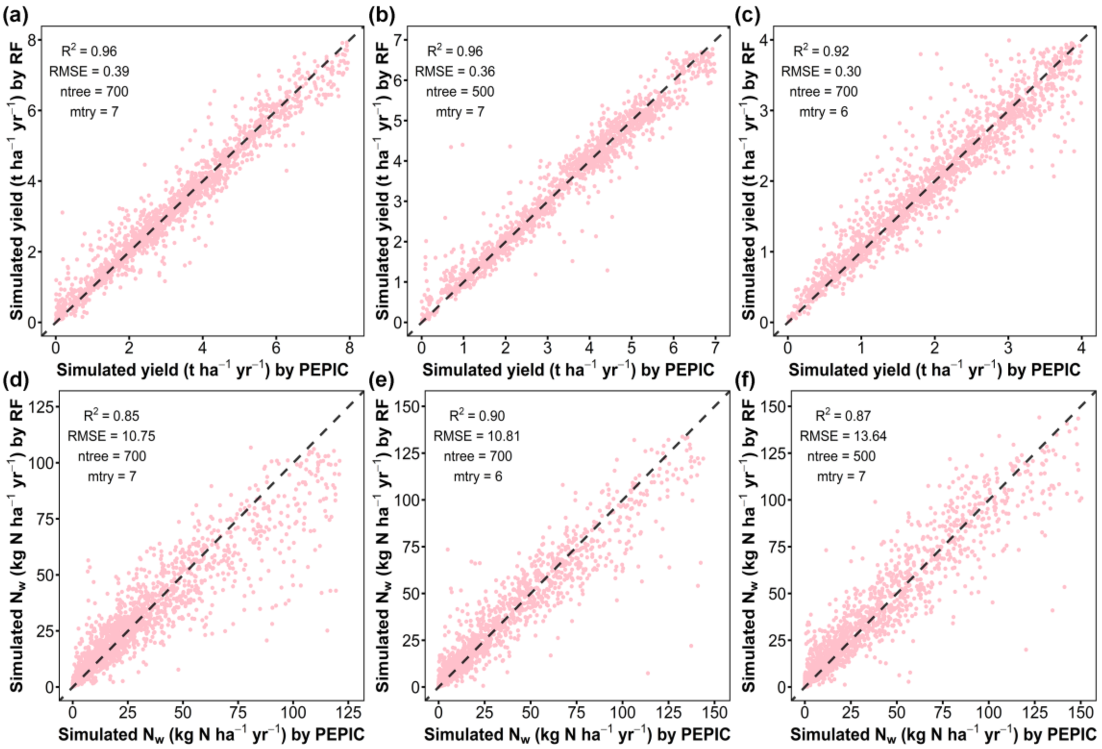

Fig. S16. Performance of Random Forest (RF) on representing PEPIC simulated crop yield and aquatic nitrogen losses (N_w). Dashed lines show 1:1 line. R^2: coefficient of determination; RMSE: root mean square error; ntree and mtry are the RF parameters. The comparison is presented for three crops: maize (a, d), rice (b, e), and wheat (c, f).

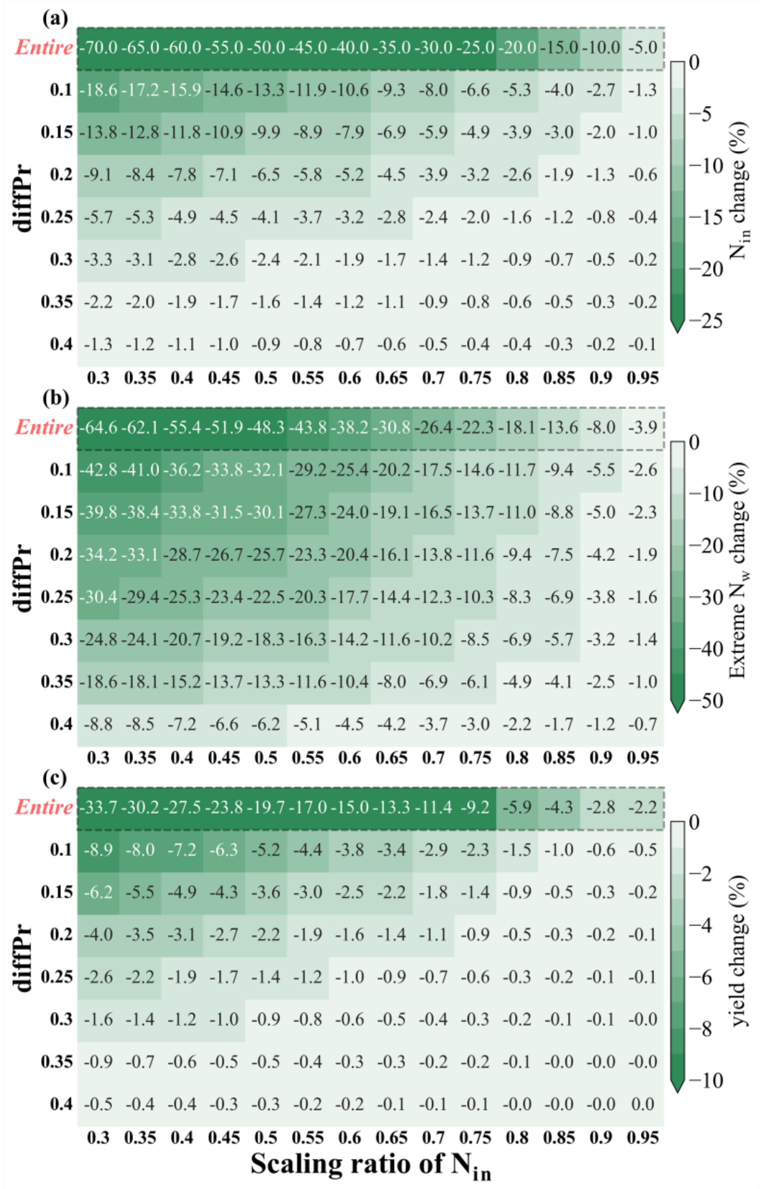

Fig. S17. Mitigation of extreme nitrogen losses (N_w). The heat maps show a) reduction in nitrogen input (N_{in}) over the 1981–2010 period, b) reduction in N_w over the extremely wet years, and c) reduction in crop yield over the 1981–2010 period resulting from a reduction of N_{in} to a certain level relative to current inputs. 'Entire' means N_{in} is scaled down over the entire period, while 'diffPr' means N_{in} is scaled down only over years with relative precipitation difference between two consecutive years (diffPr) higher than a certain threshold. Reductions in N_{in}, N_w, and crop yields are relative to their respective 1981–2010 average. The three crops under both irrigated and rainfed conditions are aggregated by area-weighted averages.

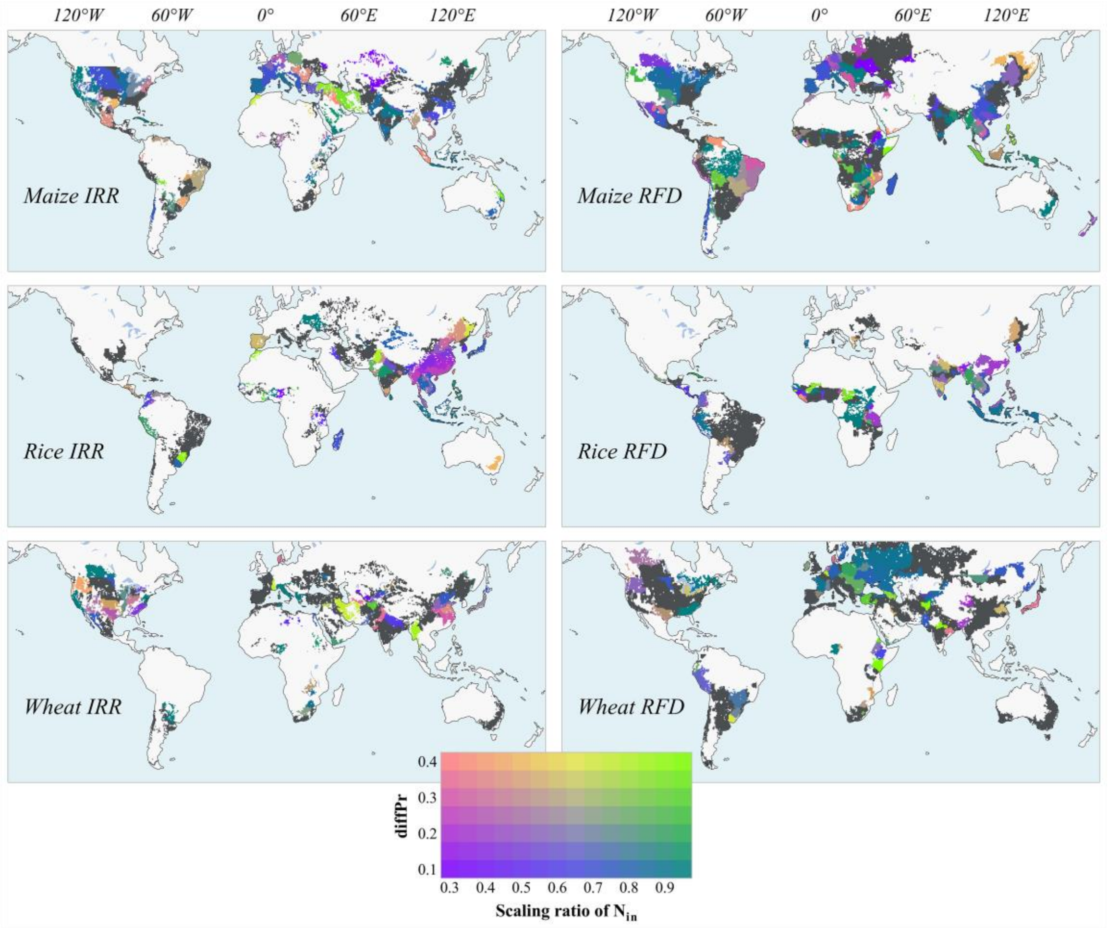

Fig. S18. Similar to Fig. 5 in the main text, but without any compromises on crop yield. Irrigated (IRR) and rainfed (RFD) are distinguished for three crops: maize, rice, and wheat.

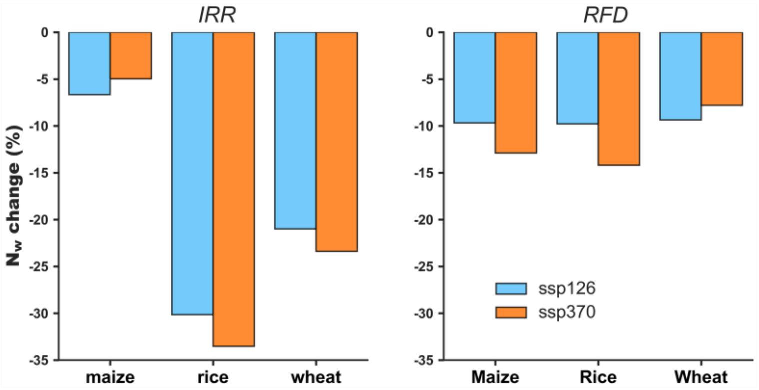

Fig. S19. Mitigation of future extreme nitrogen losses (N_w) distinguished by irrigated (IRR) and rainfed (RFD) conditions. The mitigation measures are achieved by using the diffPr thresholds and N input scaling ratios shown in fig. S18.

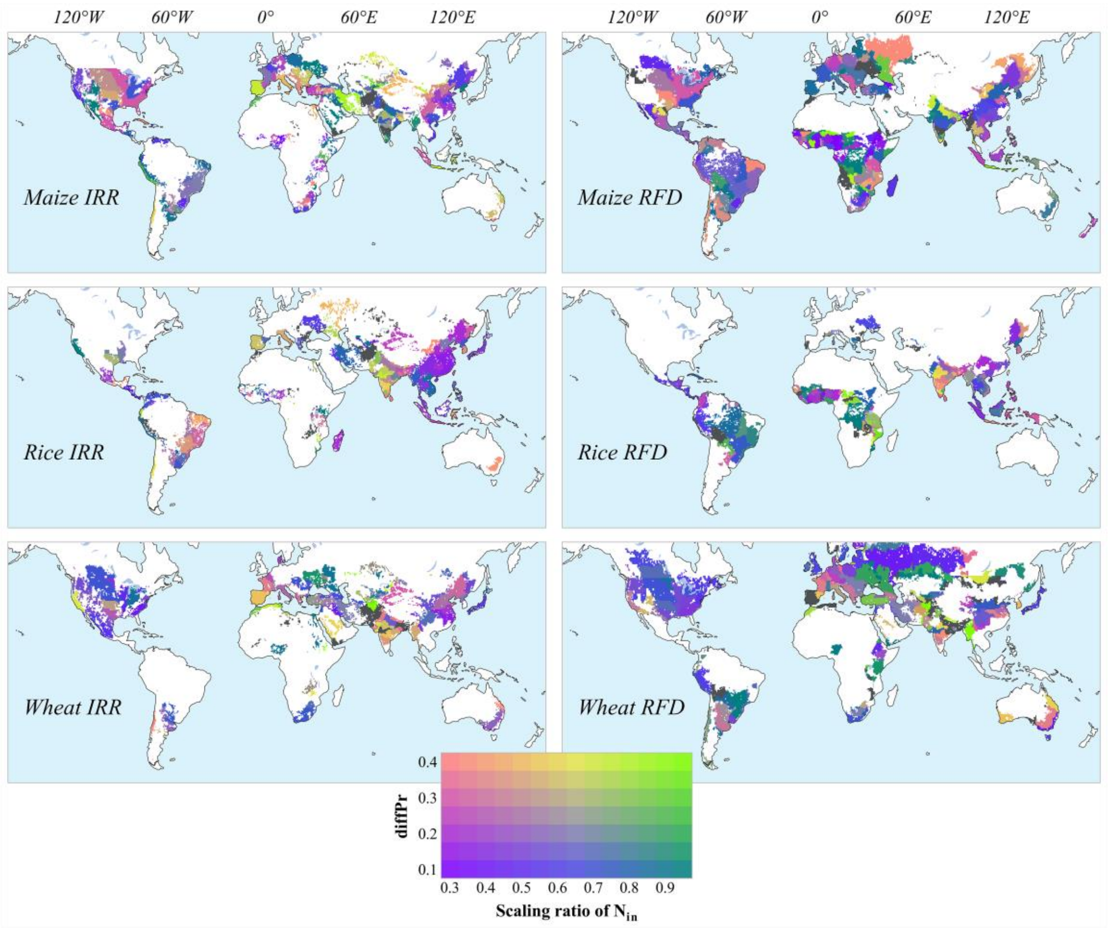

Fig. S20. Similar to Fig. 5 in the main text, but for the scenario SSP126. Irrigated (IRR) and rainfed (RFD) are distinguished for three crops: maize, rice, and wheat.

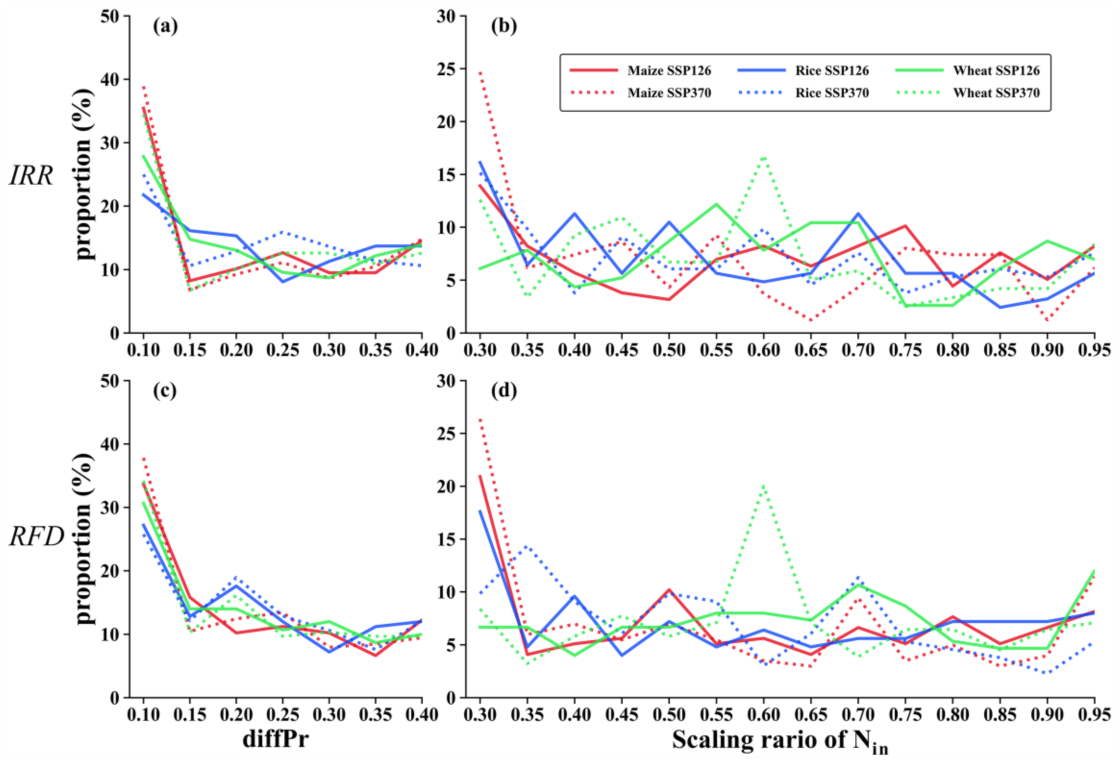

Fig. S21. Proportion of the diffPr thresholds and scaling ratios of nitrogen input of the measures in Fig. 5 in the main text. Rows are designated as IRR for irrigated areas (a, b) and RFD for rainfed areas (c, d).

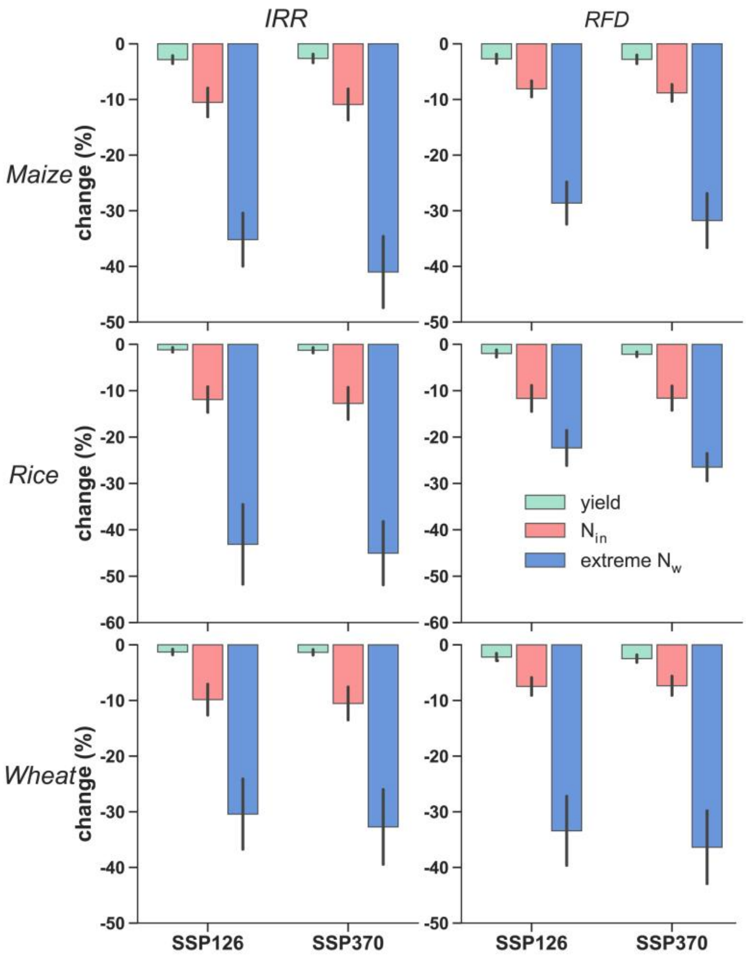

Fig. S22. Mitigation of future extreme nitrogen losses. The figure shows changes of the future extreme nitrogen losses (N_w), of the 2036–2065 average nitrogen input (N_{in}) and crop yield with mitigation vs. no-mitigation relative to the 1981–2010 averages. The mitigation is achieved through using different optimal combinations of diffPr thresholds and N_{in} scaling ratios by setting different constraints on the reductions of N_{in} (10%, 15%, and 20%) and of yield (3% and 5%). Bars show the mean changes of different constraints, while error lines are the standard deviation. Irrigated (IRR) and rainfed (RFD) are distinguished for three crops: maize, rice, and wheat.

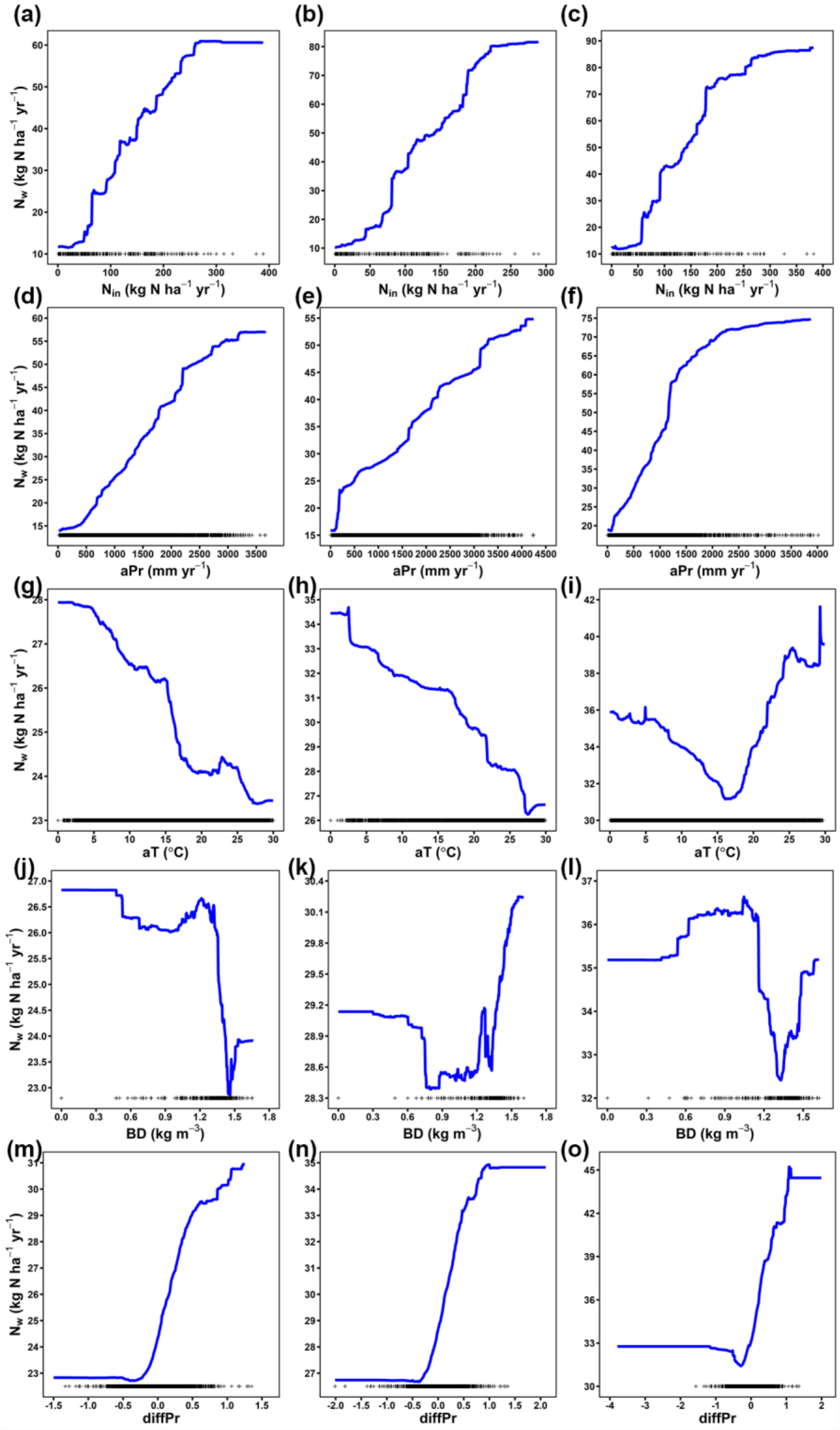

Fig. S23. One dimensional partial dependence plots of aquatic nitrogen losses (N_w) to different influencing factors according to the Random Forest simulations. N_{in}: N fertilizer inputs (a-c); aPr: annual precipitation (d-f); aT: annual temperature (g-i); DB: soil bulk density (j-l); diffPr: relative precipitation difference between two

consecutive years (m-o). The bottom rug denotes data density. Three columns represent for three crops: maize (left), rice (middle), and wheat (right).

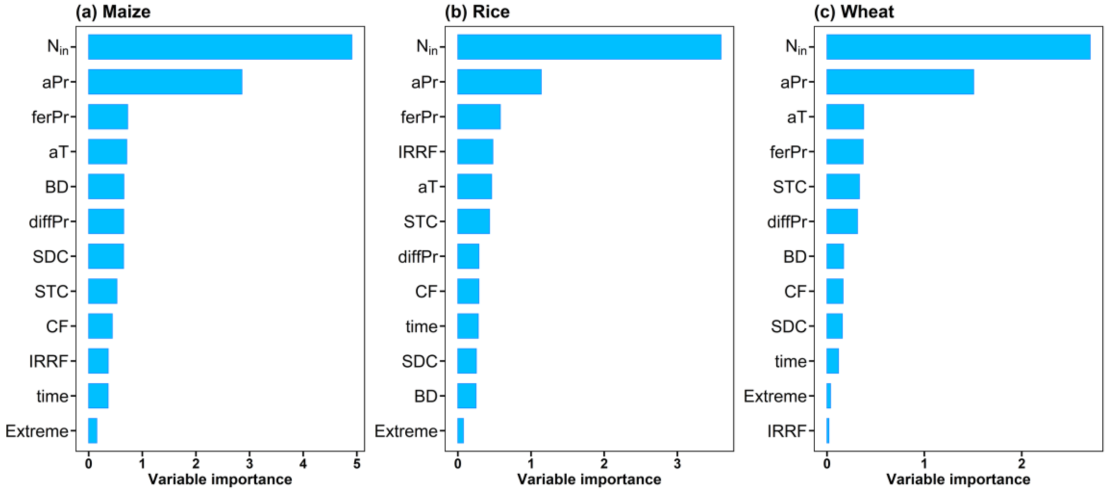

Fig. S24. Relative importance of the Random Forest models for simulating aquatic nitrogen losses of maize (a), rice (b), and wheat (c). N_{in}: N input; aPr: annual precipitation; ferPr: precipitation during N fertilization period; aT: annual temperature; STC: silt content; CF: coarse fragment; DB: soil bulk density; SDC: sand content; diffPr: relative precipitation difference between two consecutive years; IRRF: distinguishing irrigated or rainfed cultivations; Extreme: distinguishing extremely wet years or other years.

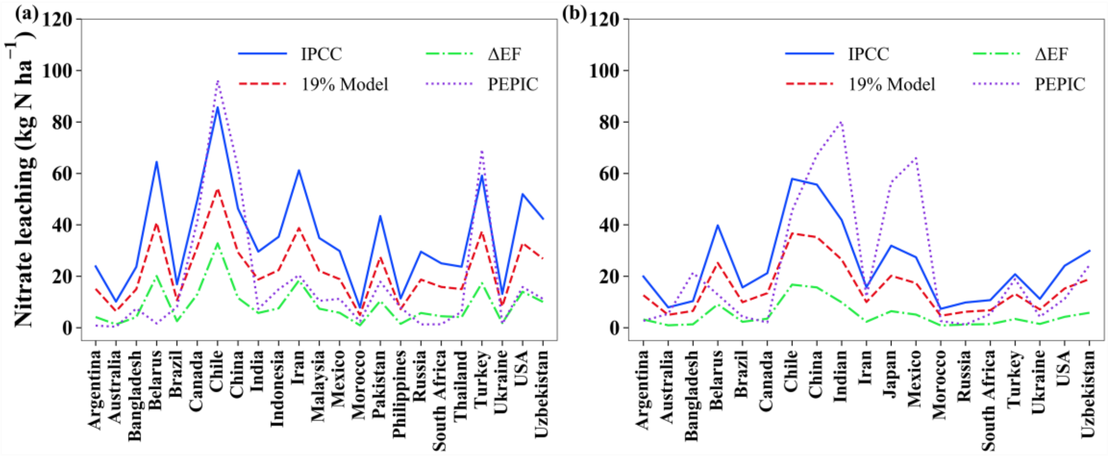

Fig. S25. Comparison of nitrate leaching for maize (a) and wheat (b) based on the four different leaching models. Soil nitrate leaching emission factor (EF) was used to estimate nitrate leaching. Nitrate leaching estimates are provided by IPCC (the International Panel on Climate Change (*71*), 30% EF), 19% Model (19% EF, Lin et al. (2001) (*72*)), ΔEF (empirical model with inconstant EF, Wang et al. (2019) (*73*)) and simulations by PEPIC.

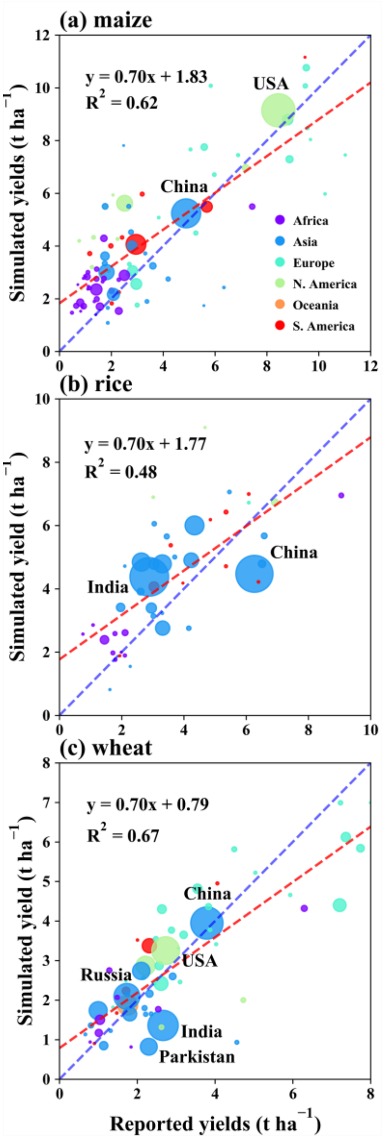

Fig. S26. Performance of PEPIC on simulating crop yield at the country level for maize (a), rice (b), and wheat (c). Equations show the linear regression between reported yields (from FAOSTAT) and simulated yields by PEPIC with coefficient of determination (R^2). Red dashed lines are the linear regression lines and blue ones are the 1:1 line. Colors represent different continents and sizes represent cropland areas for each country.

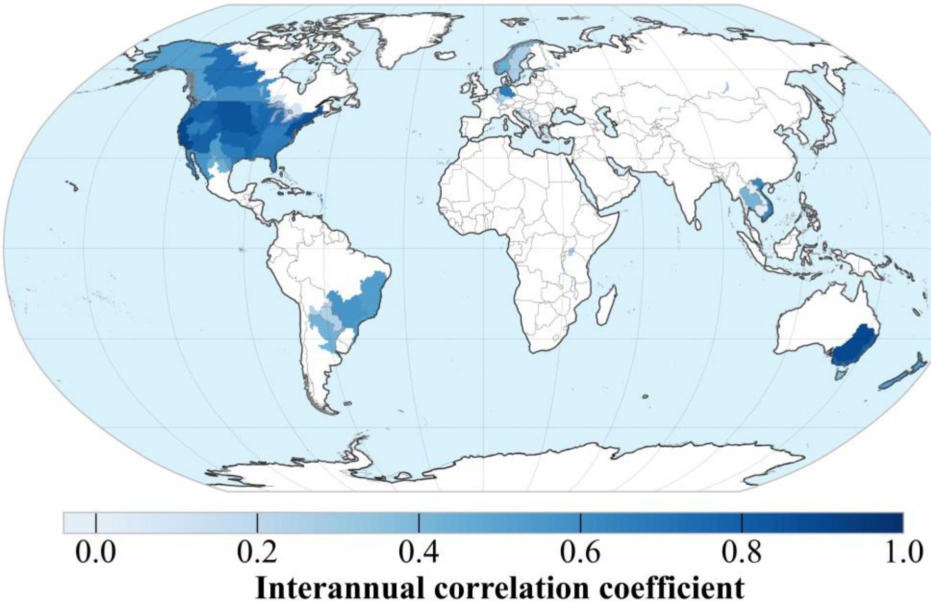

Fig. S27. Correlation between normalized observed total nitrogen (N) concentration in the water bodies and normalized aquatic nitrogen losses (N_w) simulated by the PEPIC model. Only the regions with observed total N concentration over 10 years were considered.

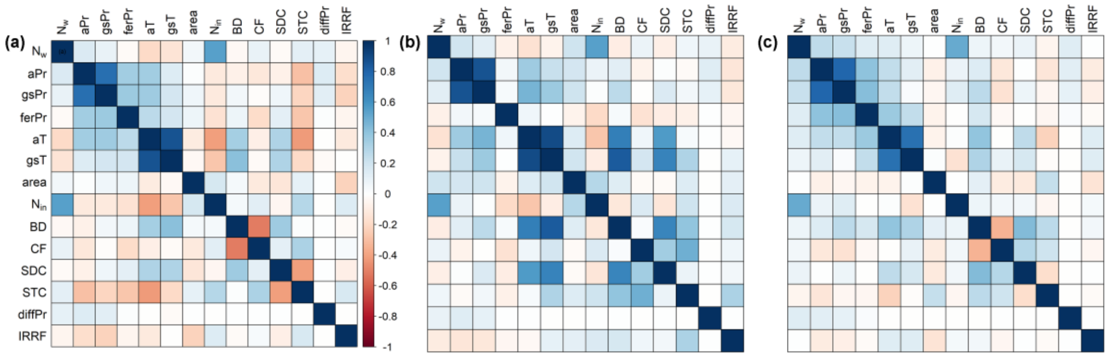

Fig. S28. Heat maps of auto-correlation of variables for maize (a), rice (b), and wheat (c). N_{in}: N input; aPr: annual precipitation; gsPr: growing season precipitation; ferPr: precipitation during N fertilization period; aT: annual temperature; gsT: growing season temperature; STC: silt content; CF: coarse fragment; DB: soil bulk density; SDC: sand content; diffPr: relative precipitation difference between two consecutive years; IRRF: irrigated or rainfed cultivations.

Table S1. Proportion of extremely wet years (EWYs) to all years in the historical and future periods. IRR: irrigated; RFD: rainfed.

	History		SSP126		SSP370	
	IRR	RFD	IRR	RFD	IRR	RFD
Maize	3.0	2.6	24.0	24.9	21.7	22.6
Rice	2.7	2.8	20.2	21.7	17.8	19.1
Wheat	3.0	2.7	25.0	27.7	24.4	26.8

Table S2. Nitrogen input (N_{in}, kg N ha^{-1} yr^{-1}) in historical and future periods under irrigated (IRR) and rainfed (RFD) cultivations. IRR: irrigated; RFD: rainfed.

	History		SSP126		SSP370	
	IRR	RFD	IRR	RFD	IRR	RFD
Maize	184.7	107.3	193.7	116.9	217.3	129.0
Rice	146.2	86.3	168.5	113.s2	173.5	97.1
Wheat	148.2	76.3	167.0	82.9	174.5	87.4

Table S3. Comparison of ratio of nitrogen losses (N_w, Tg N yr^{-1}) to nitrogen input (N_{in}, Tg N yr^{-1}).

	Bouwman et al. (2009) (*74*)	Bouwman et al. (2013) (*75*)	Liu et al. (2010) (*76*)	Mekonnen and Hoekstra (2015) (*77*)	Sutton et al. (2013) (*78*)	This study
N_{in}	184	175	85	134	177	111
N_w	41	57	47	53	95	45
N_w/N_{in}	0.22	0.33	0.55	0.4	0.54	0.41

REFERENCES AND NOTES

1. T. Hasegawa, G. Sakurai, S. Fujimori, K. Takahashi, Y. Hijioka, T. Masui, Extreme climate events increase risk of global food insecurity and adaptation needs. *Nat. Food* **2**, 587–595 (2021).

2. M. C. Kirchmeier-Young, X. Zhang, Human influence has intensified extreme precipitation in North America. *Proc. Natl. Acad. Sci.* **117**, 13308–13313 (2020).

3. P. Stott, How climate change affects extreme weather events. *Science* **352**, 1517–1518 (2016).

4. O. M. Ogega, J. Koske, J. B. Kung'u, E. Scoccimarro, H. S. Endris, M. N. Mistry, Heavy precipitation events over East Africa in a changing climate: Results from CORDEX RCMs. *Climate Dynam.* **55**, 993–1009 (2020).

5. R. D. Harp, D. E. Horton, Observed changes in daily precipitation intensity in the United States. *Geophys. Res. Lett.* **49**, e2022GL099955 (2022).

6. J. Lehmann, D. Coumou, K. Frieler, Increased record-breaking precipitation events under global warming. *Clim. Change* **132**, 501–515 (2015).

7. E. M. Fischer, R. Knutti, Observed heavy precipitation increase confirms theory and early models. *Nat. Clim. Change* **6**, 986–991 (2016).

8. J.-S. Ye, Y.-H. Gong, F. Zhang, J. Ren, X.-K. Bai, Y. Zheng, Which temperature and precipitation extremes best explain the variation of warm versus cold years and wet versus dry years? *J. Climate* **31**, 45–59 (2018).

9. C. C. Ummenhofer, G. A. Meehl, Extreme weather and climate events with ecological relevance: A review. *Philos. Trans. R. Soc. Lond. B Biol. Sci.* **372**, 20160135 (2017).

10. S. Wen, Y. Tian, S. Ouyang, M. Song, X. Li, Y. Zhang, S. Gao, X. Xu, Y. Kuzyakov, High frequency of extreme precipitation increases *Stipa grandis* biomass by altering plant and microbial nitrogen acquisition. *Biol. Fertil. Soils* **58**, 63–75 (2022).

11. E. Sinha, A. M. Michalak, V. Balaji, Eutrophication will increase during the 21st century as a result of precipitation changes. *Science* **357**, 405–408 (2017).

12. M. Lee, E. Shevliakova, S. Malyshev, P. C. D. Milly, P. R. Jaffé, Climate variability and extremes, interacting with nitrogen storage, amplify eutrophication risk. *Geophys. Res. Lett.* **43**, 7520–7528 (2016).

13. T. C. Ballard, E. Sinha, A. M. Michalak, Long-term changes in precipitation and temperature have already impacted nitrogen loading. *Environ. Sci. Technol.* **53**, 5080–5090 (2019).

14. N. W. Baeumler, S. C. Gupta, Precipitation as the primary driver of variability in river nitrogen loads in the Midwest United States. *J. Am. Water Resour. Assoc.* **56**, 113–133 (2020).

15. L. Lassaletta, A. Sanz-Cobena, E. Aguilera, M. Quemada, G. Billen, A. Bondeau, M. L. Cayuela, W. Cramer, J. P. C. Eekhout, J. Garnier, B. Grizzetti, D. S. Intrigliolo, M. R. Ramos, E. Romero, A. Vallejo, B. S. Gimeno, Nitrogen dynamics in cropping systems under Mediterranean climate: A systemic analysis. *Environ. Res. Lett.* **16**, 073002 (2021).

16. Y. Dai, S. Yang, D. Zhao, C. Hu, W. Xu, D. M. Anderson, Y. Li, X.-P. Song, D. G. Boyce, L. Gibson, C. Zheng, L. Feng, Coastal phytoplankton blooms expand and intensify in the 21st century. *Nature* **615**, 280–284 (2023).

17. B. Qin, J. Deng, K. Shi, J. Wang, J. Brookes, J. Zhou, Y. Zhang, G. Zhu, H. W. Paerl, L. Wu, Extreme climate anomalies enhancing cyanobacterial blooms in eutrophic Lake Taihu, China. *Water Resour. Res.* **57**, e2020WR029371 (2021).

18. M. J. Winnick, Stream transport and substrate controls on nitrous oxide yields from hyporheic zone denitrification. *AGU Adv.* **2**, e2021AV000517 (2021).

19. C. W. Thackeray, A. Hall, J. Norris, D. Chen, Constraining the increased frequency of global precipitation extremes under warming. *Nat. Clim. Change* **12**, 441–448 (2022).

20. W. Liu, H. Yang, C. Folberth, C. Müller, P. Ciais, K. C. Abbaspour, R. Schulin, Achieving high crop yields with low nitrogen emissions in global agricultural input intensification. *Environ. Sci. Technol.* **52**, 13782–13791 (2018).

21. C. De Notaris, J. Rasmussen, P. Sørensen, J. E. Olesen, Nitrogen leaching: A crop rotation perspective on the effect of N surplus, field management and use of catch crops. *Agric. Ecosyst. Environ.* **255**, 1–11 (2018).

22. K. Liang, X. Zhong, J. Pan, N. Huang, Y. Liu, B. Peng, Y. Fu, X. Hu, Reducing nitrogen surplus and environmental losses by optimized nitrogen and water management in double rice cropping system of South China. *Agric. Ecosyst. Environ.* **286**, 106680 (2019).

23. X. Zhang, E. A. Davidson, D. L. Mauzerall, T. D. Searchinger, P. Dumas, Y. Shen, Managing nitrogen for sustainable development. *Nature* **528**, 51–59 (2015).

24. B. Gu, X. Zhang, S. K. Lam, Y. Yu, H. J. M. van Grinsven, S. Zhang, X. Wang, B. L. Bodirsky, S. Wang, J. Duan, C. Ren, L. Bouwman, W. de Vries, J. Xu, M. A. Sutton, D. Chen, Cost-effective mitigation of nitrogen pollution from global croplands. *Nature* **613**, 77–84 (2023).

25. M. Springmann, M. Clark, D. Mason-D'Croz, K. Wiebe, B. L. Bodirsky, L. Lassaletta, W. de Vries, S. J. Vermeulen, M. Herrero, K. M. Carlson, M. Jonell, M. Troell, F. DeClerck, L. J. Gordon, R. Zurayk, P. Scarborough, M. Rayner, B. Loken, J. Fanzo, H. C. J. Godfray, D. Tilman, J. Rockström, W. Willett, Options for keeping the food system within environmental limits. *Nature* **562**, 519–525 (2018).

26. T. L. Greaver, C. M. Clark, J. E. Compton, D. Vallano, A. F. Talhelm, C. P. Weaver, L. E. Band, J. S. Baron, E. A. Davidson, C. L. Tague, E. Felker-Quinn, J. A. Lynch, J. D. Herrick, L. Liu, C. L. Goodale, K. J. Novak, R. A. Haeuber, Key ecological responses to nitrogen are altered by climate change. *Nat. Clim. Change* **6**, 836–843 (2016).

27. T. M. Bowles, S. S. Atallah, E. E. Campbell, A. C. M. Gaudin, W. R. Wieder, A. S. Grandy, Addressing agricultural nitrogen losses in a changing climate. *Nat. Sustain.* **1**, 399–408 (2018).

28. W. Liu, H. Yang, J. Liu, L. B. Azevedo, X. Wang, Z. Xu, K. C. Abbaspour, R. Schulin, Global assessment of nitrogen losses and trade-offs with yields from major crop cultivations. *Sci. Total Environ.* **572**, 526–537 (2016).

29. C. Müller, J. Elliott, J. Chryssanthacopoulos, A. Arneth, J. Balkovic, P. Ciais, D. Deryng, C. Folberth, M. Glotter, S. Hoek, T. Iizumi, R. C. Izaurralde, C. Jones, N. Khabarov, P. Lawrence, W. Liu, S. Olin, T. A. M. Pugh, D. K. Ray, A. Reddy, C. Rosenzweig, A. C. Ruane, G. Sakurai, E. Schmid, R. Skalsky, C. X. Song, X. Wang, A. de Wit, H. Yang, Global gridded crop model evaluation: Benchmarking, skills, deficiencies and implications. *Geosci. Model Dev.* **10**, 1403–1422 (2017).

30. X. Cai, M. W. Rosegrant, Global water demand and supply projection: Part 1. A modeling approach. *Water Int.* **27**, 159–169 (2002).

31. M. Kummu, P. J. Ward, H. de Moel, O. Varis, Is physical water scarcity a new phenomenon? Global assessment of water shortage over the last two millennia. *Environ. Res. Lett.* **5**, 034006 (2010).

32. B. C. O'Neill, C. Tebaldi, D. P. van Vuuren, V. Eyring, P. Friedlingstein, G. Hurtt, R. Knutti, E. Kriegler, J.-F. Lamarque, J. Lowe, G. A. Meehl, R. Moss, K. Riahi, B. M. Sanderson, The Scenario Model Intercomparison Project (ScenarioMIP) for CMIP6. *Geosci. Model Dev.* **9**, 3461–3482 (2016).

33. T. K. Ho, "Random decision forests" in *Proceedings of 3rd International Conference on Document Analysis and Recognition* (IEEE, 1995), vol. 1, pp. 278–282.

34. D. R. Kanter, W. Winiwarter, B. L. Bodirsky, L. Bouwman, E. Boyer, S. Buckle, J. E. Compton, T. Dalgaard, W. de Vries, D. Leclère, A. Leip, C. Müller, A. Popp, N. Raghuram,

S. Rao, M. A. Sutton, H. Tian, H. Westhoek, X. Zhang, M. Zurek, A framework for nitrogen futures in the shared socioeconomic pathways. *Glob. Environ. Change* **61**, 102029 (2020).

35. B. Z. Houlton, M. Almaraz, V. Aneja, A. T. Austin, E. Bai, K. G. Cassman, J. E. Compton, E. A. Davidson, J. W. Erisman, J. N. Galloway, B. Gu, G. Yao, L. A. Martinelli, K. Scow, W. H. Schlesinger, T. P. Tomich, C. Wang, X. Zhang, A world of cobenefits: Solving the global nitrogen challenge. *Earths Future* **7**, 865–872 (2019).

36. E. Sinha, A. M. Michalak, V. Balaji, L. Resplandy, India's riverine nitrogen runoff strongly impacted by monsoon variability. *Environ. Sci. Technol.* **56**, 11335–11342 (2022).

37. W. Liu, P. Ciais, X. Liu, H. Yang, A. Y. Hoekstra, Q. Tang, X. Wang, X. Li, L. Cheng, Global phosphorus losses from croplands under future precipitation scenarios. *Environ. Sci. Technol.* **54**, 14761–14771 (2020).

38. T. D. Loecke, A. J. Burgin, D. A. Riveros-Iregui, A. S. Ward, S. A. Thomas, C. A. Davis, M. A. St Clair, Weather whiplash in agricultural regions drives deterioration of water quality. *Biogeochemistry* **133**, 7–15 (2017).

39. N. D. Mueller, J. S. Gerber, M. Johnston, D. K. Ray, N. Ramankutty, J. A. Foley, Closing yield gaps through nutrient and water management. *Nature* **490**, 254–257 (2012).

40. X. Chen, Z. Cui, M. Fan, P. Vitousek, M. Zhao, W. Ma, Z. Wang, W. Zhang, X. Yan, J. Yang, X. Deng, Q. Gao, Q. Zhang, S. Guo, J. Ren, S. Li, Y. Ye, Z. Wang, J. Huang, Q. Tang, Y. Sun, X. Peng, J. Zhang, M. He, Y. Zhu, J. Xue, G. Wang, L. Wu, N. An, L. Wu, L. Ma, W. Zhang, F. Zhang, Producing more grain with lower environmental costs. *Nature* **514**, 486–489 (2014).

41. S. Cai, X. Zhao, C. M. Pittelkow, M. Fan, X. Zhang, X. Yan, Optimal nitrogen rate strategy for sustainable rice production in China. *Nature* **615**, 73–79 (2023).

42. D. Wuepper, S. Le Clech, D. Zilberman, N. Mueller, R. Finger, Countries influence the trade-off between crop yields and nitrogen pollution. *Nat. Food* **1**, 713–719 (2020).

43. J. Penuelas, F. Coello, J. Sardans, A better use of fertilizers is needed for global food security and environmental sustainability. *Agric. Food Secur.* **12**, 5 (2023).

44. M. A. Sutton, C. M. Howard, D. R. Kanter, L. Lassaletta, A. Móring, N. Raghuram, N. Read, The nitrogen decade: Mobilizing global action on nitrogen to 2030 and beyond. *One Earth* **4**, 10–14 (2021).

45. J. Schewe, S. N. Gosling, C. Reyer, F. Zhao, P. Ciais, J. Elliott, L. Francois, V. Huber, H. K. Lotze, S. I. Seneviratne, M. T. H. van Vliet, R. Vautard, Y. Wada, L. Breuer, M. Büchner, D. A. Carozza, J. Chang, M. Coll, D. Deryng, A. de Wit, T. D. Eddy, C. Folberth, K. Frieler, A. D. Friend, D. Gerten, L. Gudmundsson, N. Hanasaki, A. Ito, N. Khabarov, H. Kim, P. Lawrence, C. Morfopoulos, C. Müller, H. M. Schmied, R. Orth, S. Ostberg, Y. Pokhrel, T. A. M. Pugh, G. Sakurai, Y. Satoh, E. Schmid, T. Stacke, J. Steenbeek, J. Steinkamp, Q. Tang, H. Tian, D. P. Tittensor, J. Volkholz, X. Wang, L. Warszawski, State-of-the-art global models underestimate impacts from climate extremes. *Nat. Commun.* **10**, 1005 (2019).

46. W. Zhang, K. Furtado, T. Zhou, P. Wu, X. Chen, Constraining extreme precipitation projections using past precipitation variability. *Nat. Commun.* **13**, 6319 (2022).

47. Z. Yin, B. Zhou, M. Duan, H. Chen, H. Wang, Climate extremes become increasingly fierce in China. *Innovation* **4**, 100406 (2023).

48. J. C. Ho, A. M. Michalak, N. Pahlevan, Widespread global increase in intense lake phytoplankton blooms since the 1980s. *Nature* **574**, 667–670 (2019).

49. J. Zhang, C. Lu, H. Feng, D. Hennessy, Y. Guan, M. M. Wright, Extreme climate increased crop nitrogen surplus in the United States. *Agric. For. Meteorol.* **310**, 108632 (2021).

50. H. Chen, Q. Zhang, H. Cai, W. Zhou, F. Xu, $H_2 O_2$ mediates nitrate-induced iron chlorosis by regulating iron homeostasis in rice. *Plant Cell Environ.* **41**, 767–781 (2018).

51. P. Chen, J. Xu, Z. Zhang, T. Nie, “Preferential” ammonium uptake by rice does not always turn into higher N recovery of fertilizer sources under water-saving irrigation. *Agric Water Manag* **272**, 107867 (2022).

52. P. Potter, N. Ramankutty, E. M. Bennett, S. D. Donner, Characterizing the spatial patterns of global fertilizer application and manure production. *Earth Interact.* **14**, 1–22 (2010).

53. C. Lu, H. Tian, Global nitrogen and phosphorus fertilizer use for agriculture production in the past half century: Shifted hot spots and nutrient imbalance. *Earth Syst. Sci. Data* **9**, 181–192 (2017).

54. J. N. Galloway, A. R. Townsend, J. W. Erisman, M. Bekunda, Z. Cai, J. R. Freney, L. A. Martinelli, S. P. Seitzinger, M. A. Sutton, Transformation of the nitrogen cycle: Recent trends, questions, and potential solutions. *Science* **320**, 889–892 (2008).

55. W. Liu, H. Yang, C. Folberth, X. Wang, Q. Luo, R. Schulin, Global investigation of impacts of PET methods on simulating crop-water relations for maize. *Agric. For. Meteorol.* **221**, 164–175 (2016).

56. H. Virro, G. Amatulli, A. Kmoch, L. Shen, E. Uuemaa, GRQA: Global River Water Quality archive. *Earth Syst. Sci. Data* **13**, 5483–5507 (2021).

57. J. Iqbal, M. Necpalova, S. V. Archontoulis, R. P. Anex, M. Bourguignon, D. Herzmann, D. C. Mitchell, J. E. Sawyer, Q. Zhu, M. J. Castellano, Extreme weather-year sequences have nonadditive effects on environmental nitrogen losses. *Glob. Change Biol.* **24**, e303–e317 (2018).

58. Y. Li, K. Guan, G. D. Schnitkey, E. DeLucia, B. Peng, Excessive rainfall leads to maize yield loss of a comparable magnitude to extreme drought in the United States. *Glob. Change Biol.* **25**, 2325–2337 (2019).

59. W. J. Dixon, Analysis of Extreme Values. *Ann. Math. Stat.* **21**, 488–506 (1950).

60. J. H. Friedman, Greedy function approximation: A gradient boosting machine. *Ann. Stat.* **29**, 1189–1232 (2001).

61. A. C. Ruane, R. Goldberg, J. Chryssanthacopoulos, Climate forcing datasets for agricultural modeling: Merged products for gap-filling and historical climate series estimation. *Agric. For. Meteorol.* **200**, 233–248 (2015).

62. Q. Yu, L. You, U. Wood-Sichra, Y. Ru, A. K. B. Joglekar, S. Fritz, W. Xiong, M. Lu, W. Wu, P. Yang, A cultivated planet in 2010 – Part 2: The global gridded agricultural-production maps. *Earth Syst. Sci. Data* **12**, 3545–3572 (2020).

63. W. J. Sacks, D. Deryng, J. A. Foley, N. Ramankutty, Crop planting dates: An analysis of global patterns. *Glob. Ecol. Biogeogr.* **19**, 607–620 (2010).

64. P. C. West, J. S. Gerber, P. M. Engstrom, N. D. Mueller, K. A. Brauman, K. M. Carlson, E. S. Cassidy, M. Johnston, G. K. MacDonald, D. K. Ray, S. Siebert, Leverage points for improving global food security and the environment. *Science* **345**, 325–328 (2014).

65. N. Guilpart, T. Iizumi, D. Makowski, Data-driven projections suggest large opportunities to improve Europe's soybean self-sufficiency under climate change. *Nat. Food* **3**, 255–265 (2022).

66. K. M. Carlson, J. S. Gerber, N. D. Mueller, M. Herrero, G. K. MacDonald, K. A. Brauman, P. Havlik, C. S. O'Connell, J. A. Johnson, S. Saatchi, P. C. West, Greenhouse gas emissions intensity of global croplands. *Nat. Clim. Change* **7**, 63–68 (2017).

67. X. Zhang, T. Zou, L. Lassaletta, N. D. Mueller, F. N. Tubiello, M. D. Lisk, C. Lu, R. T. Conant, C. D. Dorich, J. Gerber, H. Tian, T. Bruulsema, T. M. Maaz, K. Nishina, B. L. Bodirsky, A. Popp, L. Bouwman, A. Beusen, J. Chang, P. Havlík, D. Leclère, J. G. Canadell, R. B. Jackson, P. Heffer, N. Wanner, W. Zhang, E. A. Davidson, Quantification of global and national nitrogen budgets for crop production. *Nat. Food* **2**, 529–540 (2021).

68. G. C. Hurtt, L. Chini, R. Sahajpal, S. Frolking, B. L. Bodirsky, K. Calvin, J. C. Doelman, J. Fisk, S. Fujimori, K. K. Goldewijk, T. Hasegawa, P. Havlik, A. Heinimann, F. Humpenöder, J. Jungclaus, J. O. Kaplan, J. Kennedy, T. Krisztin, D. Lawrence, P. Lawrence, L. Ma, O. Mertz, J. Pongratz, A. Popp, B. Poulter, K. Riahi, E. Shevliakova, E. Stehfest, P. Thornton, F.

N. Tubiello, D. P. van Vuuren, X. Zhang, Harmonization of global land use change and management for the period 850–2100 (LUH2) for CMIP6. *Geosci. Model Dev.* **13**, 5425–5464 (2020).

69. E. Sinha, A. M. Michalak, K. V. Calvin, P. J. Lawrence, Societal decisions about climate mitigation will have dramatic impacts on eutrophication in the 21st century. *Nat. Commun.* **10**, 939 (2019).

70. N. H. Batjes, ISRIC-WISE derived soil properties on a 5 by 5 arc-minutes global grid (version 1.1) (ISRIC, 2006), 55 pp.

71. IPCC, "N_2O Emissions From Managed Soils, and CO_2 Emissions From Lime and Urea Application" in *2006 IPCC Guidelines for National Greenhouse Gas Inventories* (IPCC, 2006), vol. 4, chap. 11.

72. B.L. Lin, A. Sakoda, R. Shibasaki, M. Suzuki, A modelling approach to global nitrate leaching caused by anthropogenic fertilisation. *Water Res.* **35**, 1961–1968 (2001).

73. Y. Wang, H. Ying, Y. Yin, H. Zheng, Z. Cui, Estimating soil nitrate leaching of nitrogen fertilizer from global meta-analysis. *Sci. Total Environ.* **657**, 96–102 (2019).

74. A. F. Bouwman, A. H. W. Beusen, G. Billen, Human alteration of the global nitrogen and phosphorus soil balances for the period 1970–2050. *Global Biogeochem. Cycles* **23**, 10.1029/2009GB003576 (2009).

75. L. Bouwman, K. K. Goldewijk, K. W. Van Der Hoek, A. H. W. Beusen, D. P. Van Vuuren, J. Willems, M. C. Rufino, E. Stehfest, Exploring global changes in nitrogen and phosphorus cycles in agriculture induced by livestock production over the 1900–2050 period. *Proc. Natl. Acad. Sci. U.S.A.* **110**, 20882–20887 (2013).

76. J. Liu, L. You, M. Amini, M. Obersteiner, M. Herrero, A. J. B. Zehnder, H. Yang, A high-resolution assessment on global nitrogen flows in cropland. *Proc. Natl. Acad. Sci. U.S.A.* **107**, 8035–8040 (2010).

77. M. M. Mekonnen, A. Y. Hoekstra, Global gray water footprint and water pollution levels related to anthropogenic nitrogen loads to fresh water. *Environ. Sci. Technol.* **49**, 12860–12868 (2015).

78. United Nations Environment Programme, Global Partnership on Nutrient Management, International Nitrogen Initiative, *Our nutrient world: The challenge to produce more food and energy with less pollution* (Centre for Ecology & Hydrology, 2013).
